# Practical Fundamentals of Clinical MEG Interpretation in Epilepsy

**DOI:** 10.3389/fneur.2021.722986

**Published:** 2021-10-14

**Authors:** Christopher Laohathai, John S. Ebersole, John C. Mosher, Anto I. Bagić, Ai Sumida, Gretchen Von Allmen, Michael E. Funke

**Affiliations:** ^1^Division of Child Neurology, Department of Pediatrics, McGovern Medical School at UTHealth, Houston, TX, United States; ^2^Department of Neurology, Saint Louis University, Saint Louis, MO, United States; ^3^Northeast Regional Epilepsy Group, Atlantic Health Neuroscience Institute, Summit, NJ, United States; ^4^Department of Neurology, McGovern Medical School at UTHealth, Houston, TX, United States; ^5^University of Pittsburgh Comprehensive Epilepsy Center (UPCEC), Department of Neurology, University of Pittsburgh Medical Center, Pittsburg, PA, United States

**Keywords:** magnetoencephalography, magnetic source imaging, equivalent current dipole, epilepsy, epilepsy surgery

## Abstract

Magnetoencephalography (MEG) is a neurophysiologic test that offers a functional localization of epileptic sources in patients considered for epilepsy surgery. The understanding of clinical MEG concepts, and the interpretation of these clinical studies, are very involving processes that demand both clinical and procedural expertise. One of the major obstacles in acquiring necessary proficiency is the scarcity of fundamental clinical literature. To fill this knowledge gap, this review aims to explain the basic practical concepts of clinical MEG relevant to epilepsy with an emphasis on single equivalent dipole (sECD), which is one the most clinically validated and ubiquitously used source localization method, and illustrate and explain the regional topology and source dynamics relevant for clinical interpretation of MEG-EEG.

## Introduction

Epilepsy surgery continues to not only be a necessity, but the most effective option for many patients with drug resistant epilepsy (DRE) (1). The availability of these procedures has grown, as reflected by the expansion of National Association of Epilepsy Centers (NAEC) accredited epilepsy centers from 133 centers in 2011 (2) to 261 centers in 2021 (NAEC Webinar, April 6th, 2021). The cohort of patients who presented for presurgical evaluation has also changed, with increased representation of extratemporal epilepsy surgery (3). Extratemporal epilepsy, in comparison with temporal lobe epilepsy, has been associated with worse outcomes (1) and requires additional specific investigations. Magnetoencephalography (MEG) is one of these neurophysiologic assessments (4, 5).

MEG is a non-invasive recording of cerebral activity as reflected outside of the skull in the form of magnetic fields generated by neuronal electrical currents (6). In comparison to electroencephalography (EEG), MEG is more sensitive to tangential sources from sulci and cortical planes. As the cortical surface consists of many gyrations and fissures, simulated computation analysis suggests that MEG can record 95% of cortical activity, significantly more than EEG which is more attuned to radial sources (7). Source localization by MEG is followed by co-registration with brain MRI, which provides anatomical correlation (magnetic source imaging; MSI) (8). MEG data has been proven to show correlation with electrocorticography in specific cases (9). In a study of 69 patients with suspected neocortical epilepsy, MEG provided non-redundant information in 33% of the patients and benefited 21% of patients who received surgery (10). Post-operative seizure freedom at 12-month has been associated with both complete intracranial sampling (62 vs. 25% seizure freedom) and complete resection (88 vs. 52% seizure freedom; complete defined by ≥ 70% dipole removal) of MEG clusters (11).

However, despite the considerable growth of surgical epilepsy centers and increased representation of extratemporal epilepsy, the availability of MEG remains relatively scarce. There are only 22 American Clinical Magnetoencephalography Society (ACMEGS) affiliated centers in the United States, representing less than 17% of the total NAEC accredited epilepsy centers (12). This is likely an outcome of multiple institutional (e.g., practice setting, economic priorities, strength of epilepsy program, patient profile, available personnel) and systemic (e.g., regulatory issues, insurers landscape) factors that are incompletely understood but are strongly influenced by the deeply habituated patterns of clinical practice (12). Given limited availability, the experience in MEG analysis and interpretation has been relatively constrained to the selected institutions with pre-existing technology and experienced personnel. The initial steps toward learning clinical magnetoencephalography can prove difficult even for clinical neurophysiologists and epileptologists outside of clinically productive MEG centers. This barrier is, in part, due to the lack of appropriate basic clinical literature. We view that an accessible review on practical fundamentals of clinical MEG localization and interpretation in epilepsy is much needed to narrow this knowledge gap.

This narrative review aims to explain the basic practical concepts of clinical MEG relevant to epilepsy with an emphasis on single equivalent dipole (sECD), which is one the most clinically validated and ubiquitously used source localization method, and illustrate the regional topology and source dynamics relevant for clinical interpretation of MEG-EEG (13). The information presented is gathered through an extensive review of available literature, supplemented by clinical examples provided by the authors, and supported by clinical experience from authors with long-term experience in the clinical MEG field. The article strives to make MEG localization and interpretation in epilepsy more understandable so that readers can recognize its utilities and limitations, facilitate the learning of clinical MEG, and raise awareness of clinical MEG's relevance to epilepsy surgery.

## Basic Concepts of Clinical MEG

### Basic Concepts of Source Localization and Methodologies

Neuronal activity consists of an intracellular “primary current,” whose circuit is completed by an induced extracellular “volume current.” The extracellular volume current creates scalp potentials that can be recorded by electroencephalography (EEG). The primary current and volume current simultaneously produce a magnetic field that can be recorded by MEG. For a given primary current, the calculation of corresponding scalp potential and the external magnetic field is termed a “forward problem.” In contrast, the “inverse problem” is the modeling of the implied primary current and its source location from the recorded MEG or EEG. The goal of clinical MEG in epilepsy is predominantly aimed toward solving this inverse problem.

MEG is recorded by relatively large coils in a variety of configurations (magnetometers, axial gradiometers, or planar gradiometers) in sensor space that are coupled with superconducting quantum interference devices (SQUIDs) to detect the magnetic field. Most MEG software will automatically and implicitly handle the integration of magnetic fields passing through these coils. The primary requirement for the sensor model is the accurate registration of the patient's scalp to the MEG helmet (14).

Solving the forward problem adequately requires adequate knowledge of the patient's head geometry. In epilepsy, a patient's recent MRI acquired with an epilepsy protocol that includes a sequence showing detailed cerebral anatomy (e.g., SPGR, BRAVO, MPRAGE, MULTI-ECHO) with 1 mm thickness or less, skin to skin, is used as the basis for the head model. Assumptions about the head model is where MEG and EEG have their greatest differences. Since external magnetic fields are less affected by tissue conductivity, a MEG head model represented as a single compartment sphere fitted to the inner skull surface, or as a tessellation of just the inner skull surface is generally adequate (15). A more sophisticated head model would first tessellate the inner skull, outer skull, and scalp as mesh of interconnected triangles, which has a much more realistic appearance than simple spheres. Solving, however, the electromagnetic fields on these triangles requires a more complicated mathematical approach and software, known as the Boundary Element Method (BEM). A study of MEG in epilepsy found no differences between three spherical shells and BEM models for single focal source localization (16). EEG, in contrast, is critically sensitive to the parameters of a multi-centric sphere or to the tessellation of the skull and scalp boundaries, requiring an accurate specification of skull and scalp thickness, and the conductivity values of the brain, skull, and scalp (17, 18).

This paper will primarily address source modeling of MEG data, while considering scalp EEG data and its temporal dynamics when it assists in the interpretation of MEG results. An example of the basics of data acquisition and minimum practice standards can be found in the ACMEGS's clinical practice guideline (5). Having described the head and sensor models that are registered to the patient, we next discuss the generation and interpretation of the primary neuronal current.

### Conceptual and Practical Aspects of Equivalent Current Dipole (ECD) Modeling

MEG is a non-invasive measure of neural activity, and it is widely-assumed that this activity arises from the columnar organization of cortical gray matter. Because of the inherent distance from outside the scalp to the cortex, these models are of a macro scale, such that the term “primary current” summarizes all of the fine micro-scale features of intracellular sources, sinks, induced currents, and transmembrane currents into a single conceptual primary current that traverses up and down the cortical column (19).

Basic cortical modeling assumptions of an evoked response suggest that MEG data represent the summed post-synaptic potentials (PSP) of approximately one million pyramidal neurons (6). For an evoked response, this resultant PSP current represents approximately 10 μA flowing along an effective cortical depth of 2 mm. Accordingly, the primary current is modeled as a “current dipole” of 20 μA/mm, which is equivalently and more generally expressed as 20 nano-ampere-meters (nAm). In contrast, an epileptic spike is 5–25 times stronger, about 100 to 500 nAm, which would require a larger number of pyramidal cells. The constant value of maximum dipole moment density across mammalian species ranges from 1 to 2 nAm/mm^2^ (referred to here as Okada's Constant of 1 nAm/mm^2^) (20); therefore, we can reasonably infer that MEG measures the activity of a relatively large “patch” of cortex.

**Figure 1:**
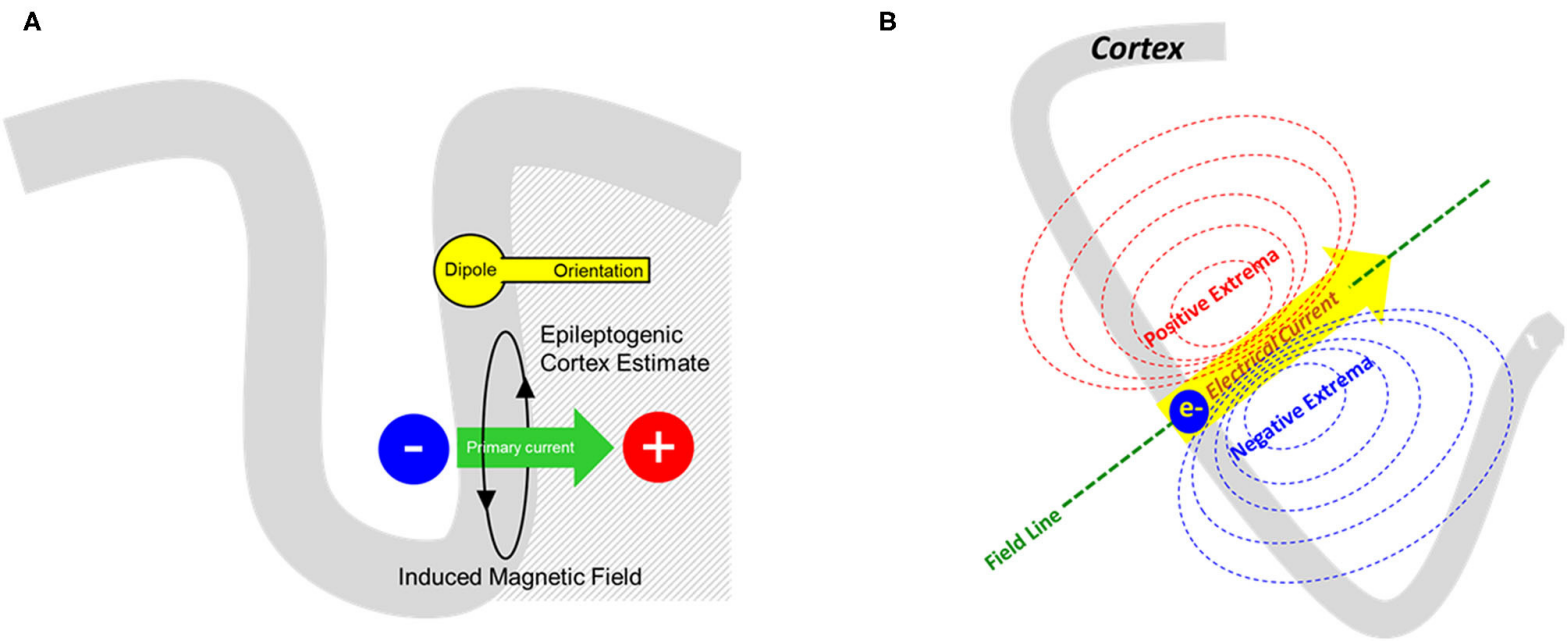
**(A)** From the measured magnetic field, an equivalent current dipole model can solve the inverse problem and provide a representation of primary current as shown here. The cortical source is represented by rounded dipole (a.k.a. “head”) whereas the direction of primary current flow (green arrow) is represented by the dipole orientation (a.k.a. “vector” or “tail”). **(B)** Schematic representation of a dipolar magnetic field pattern produced from a single electrical current source. The distance between the extremas would also signify the depth of the source in respect to the sensor.

Using this physiologic interpretation of the primary current, we can propose that the equivalent current dipole (ECD) is a simple, but plausible model for it. The ECD model represents both a source location and orientation, the latter expressing the direction of current flow (Figure 1A). In particular, the single equivalent current dipole (sECD) models the data as if it is arising from a single spot on the cortex. Six parameters define the sECD: 1) x, y, z of location, 2) azimuth and elevation orientation, and 3) dipolar strength. ACMEGS clinical practice guideline (CPG) advised that clinically relevant dipoles should have current strength between 50 and 500 nAm (21). Additional general approach to determine whether this model is indeed appropriate for measured data will be discussed later in this review.

### Selection of Discharges and Model Worthiness

**Figure 2:**
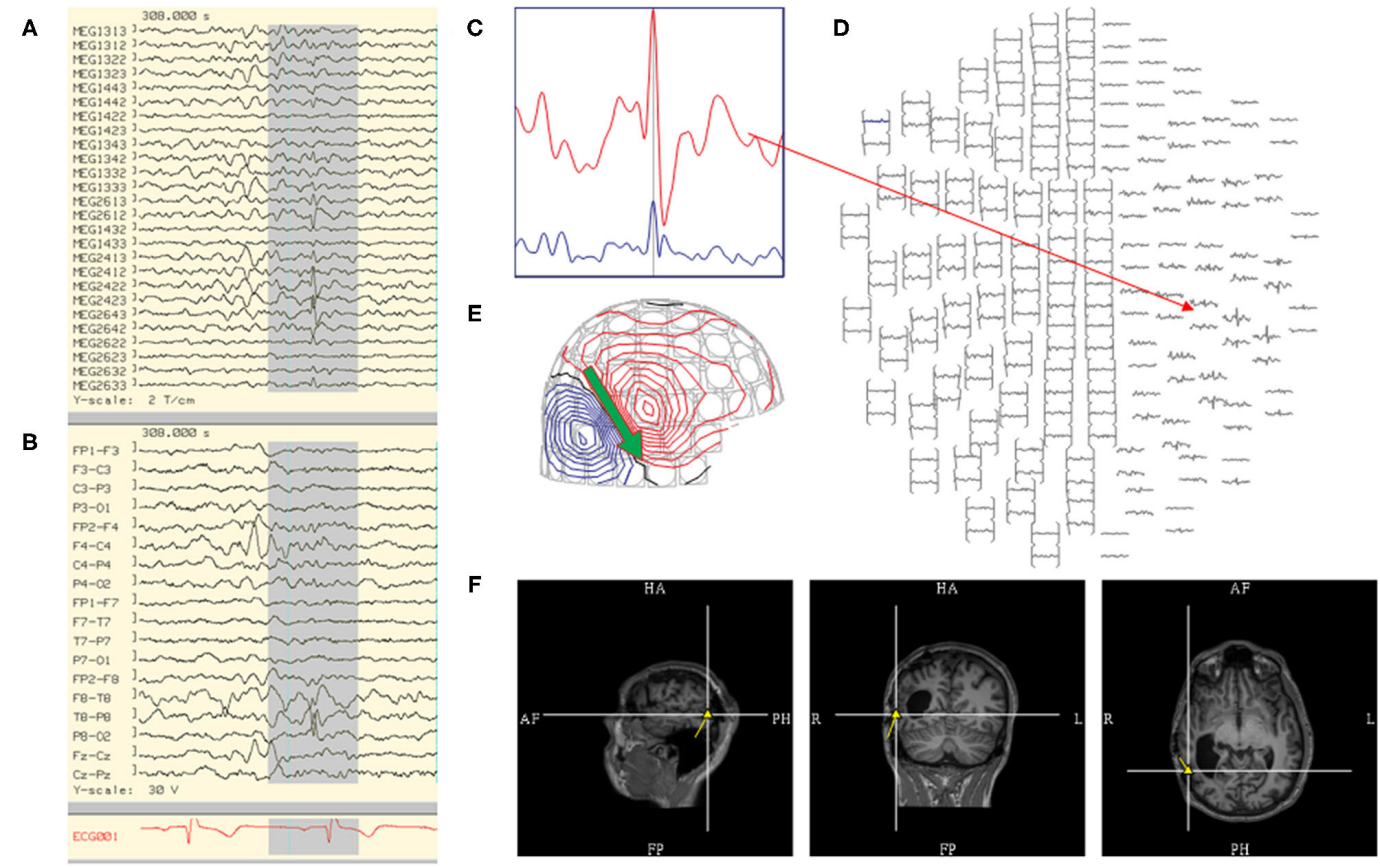
Steps in the analysis of an MEG spike include reviewing: **(A)** MEG tracing, **(B)** EEG tracing, **(C)** MEG signal selection from **(D)** magnetic isofield map, **(E)** dipolar electro-magnetic field pattern where the primary current is represented by an arrow (green), and **(F)** localization through sECD method yielding a dipole model (yellow) that is coregistered to MRI.

The selection of MEG discharges for modeling is a multi-faceted approach, involving three fundamental components: 1) waveform morphology, 2) corresponding magnetic field, and 3) anatomical localization. One approach is to first determine that a waveform is epileptiform, i.e., spike like, followed by confirming that its field is appropriately dipolar, and finally ensuring that the dipole solution is localized near an appropriately oriented cortex (Figure 2). However, these three concepts have many finer points, which will be covered in this section. The criteria for general acceptance of individual dipoles, commonly termed “fitting,” will be discussed in a separate segment.

**Figure 3:**
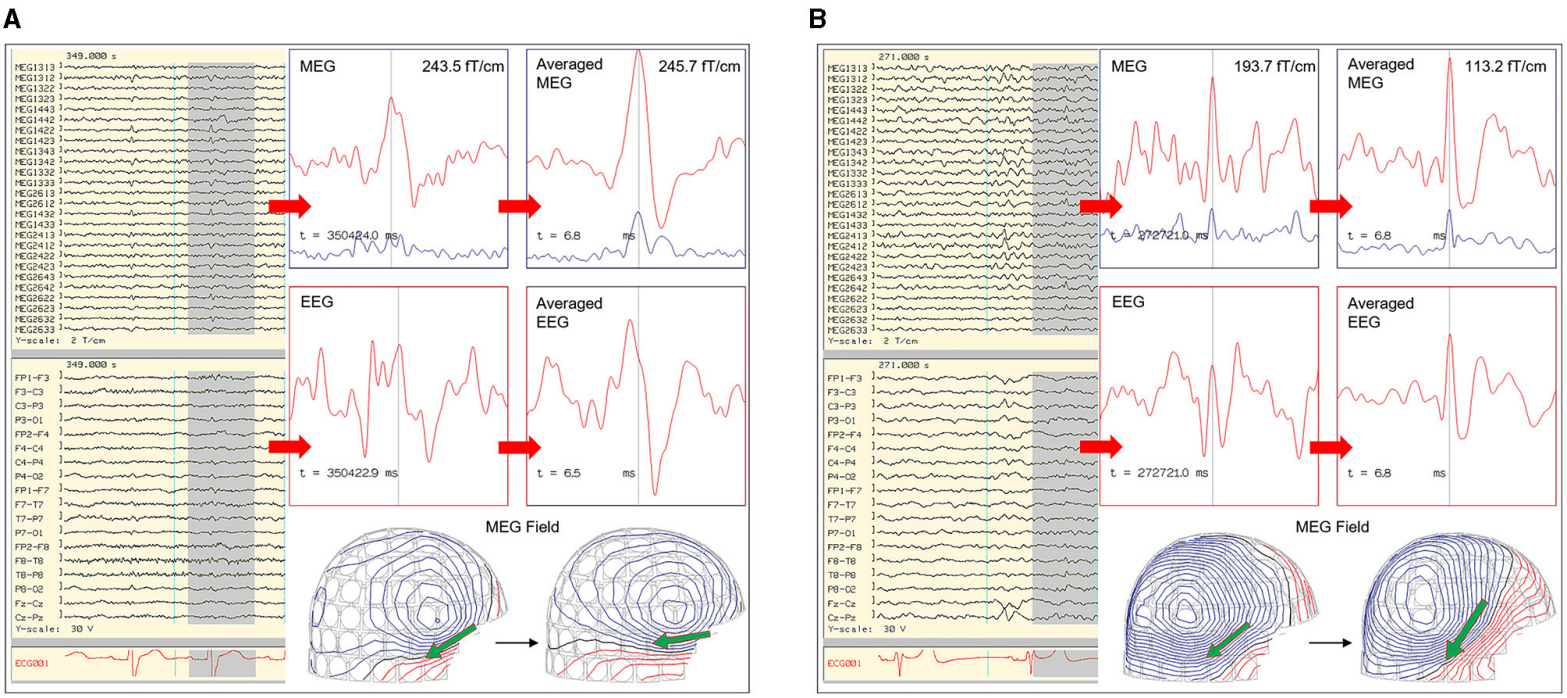
Utilization of averaging to increase signal to noise for signal detection and modeling. **(A)** MEG-EEG recording that showed MEG signals (channel MEG1442, MEG2612) with a right temporal isofield that lacked an epileptiform morphology and EEG correlate. The averaged MEG signals showed a better epileptiform morphology and EEG correlate. **(B)** MEG-EEG recording that showed a small right hemispheric EEG signal with a comparatively less visible MEG correlate, having a right temporal isofield. Averaged EEG and MEG signals show an improved epileptiform morphology.

By standard consensus, typical waveform morphologies that favor ECD modeling are those that fit the definition of traditional spikes and sharp waves (21). It has been found that MEG spikes have a tendency to have a shorter duration and sharper morphology than simultaneously recorded EEG waveforms (22). MEG spikes had a duration in range of 27–120 ms when correlated with simultaneous intracranial recording (23). However, in the same way that focal slowing in EEG may reflect underlying epileptic activity (24), MEG signals are subjected to noise which can decrease visibility of epileptic waveform and its magnetic isofield contour map. For waveforms that are potentially epileptiform but not suitable for individual modeling due to small peak magnitude, signal averaging is a method that increases the signal to noise ratio (SNR) resulting in increased visibility (21, 25) (Figure 3). Although methods can differ across laboratories, the authors average only waveforms with similar magnetic isofield locations and morphology, and similar electro-magnetic field patterns. This prevents dipole mislocalization if multiple sources are present, and avoids signal cancellation that can occur especially with intra-sulcal sources. The disadvantage of averaging is that it can be complex, and require experience.

An important practice to follow in averaging is consistency of waveform selection. In order to increase the SNR of a spike type accurately, selection of the averaging trigger point must be constant, such as the waveform's peak or a given point on its rising phase. The number of waveforms required for optimal averaging is patient dependent. A study in one patient, which included analysis of MEG averaging, showed that signal-to-noise of epileptic activity does not increase in the same way as an evoked response, but still exhibited a significant increase with averaging, and noise bias was resolved after averaging of 10 spikes (26).

After a waveform is selected, its field pattern must be evaluated prior to modeling. Important points to consider are the number of polarities, distance between extremas, and the spatio-temporal progression. A single focal source best suited for sECD modeling should produce one distinct dipolar field (Figure 1B). If a source shows multiple polarities, one can attempt to identify an early or dominant magnetic field that is consistently present, or consider other methods such as multiple ECDs or extended source modeling methods. It must be noted that basal sources close to the edge of sensor array can be less conspicuous. Extremas in close proximity would also suggest that the source is close to the sensors.

**Figure 4:**
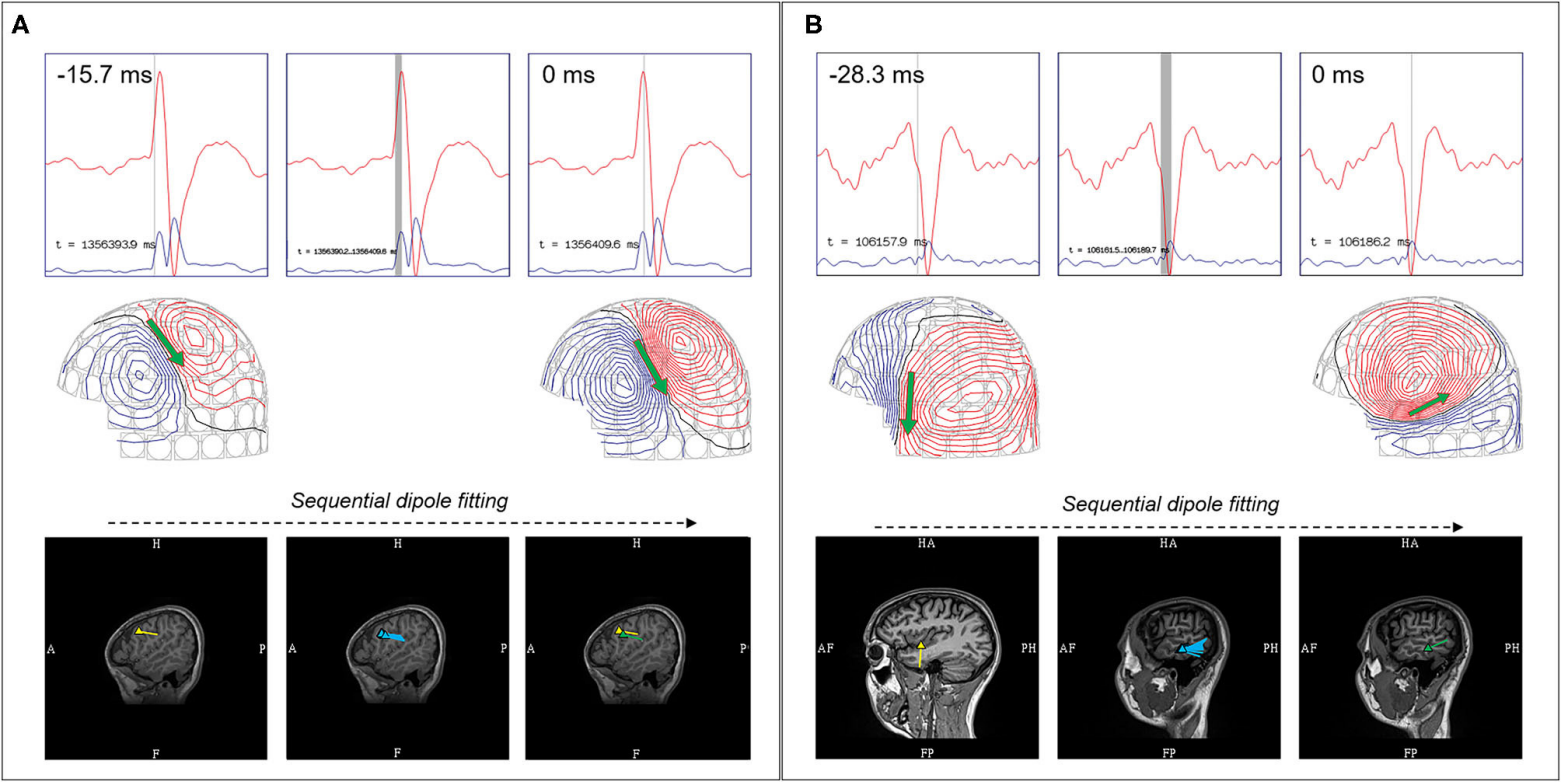
Comparison between stable and propagating sources. **(A)** Stable source with earliest localizing dipole (yellow), sequential dipoles (blue), and dipole at MEG peak (green), showed similar sublobar localization. **(B)** Propagating sources showed earliest localizing dipole (yellow) over the left mid-superior temporal gyrus, but sequential dipoles (blue) and dipole at MEG peak (green) showed propagation to the posterior middle temporal gyrus.

Spatio-temporal progression of a magnetic field during a waveform's rise to peak must also be evaluated (21). A stable source would exhibit a near-constant field pattern throughout its time course from rise to peak, typically with increasing field strength. The modeling of these sources at the peak is comparatively reliable, and signal peaks are often selected by some practitioners for ease of averaging. However, there are some signals that exhibit rotation and progression from rise to peak, which suggests a propagating pattern (Figure 4).

Finally, localization is one of the critical elements of a dipole's “model worthiness” (21). Recent observations have concluded that dipoles localized to peri-Sylvian, supramarginal, and peri-Rolandic regions frequently represent benign MEG variants (27). Epileptic semiology, imaging data, corresponding EEG findings, and overall MEG localization need to be considered for the interpretation of dipoles localized to these regions. Clinical interpretation though integration of multiple data can be complex, and is further detailed in the latter part of this review.

### General Approach to Acceptance and Interpretation of Individual Dipoles

MEG software programs can always “fit” dipoles to the data at any particular time; however, the fit will generally be poor or unacceptable by statistical standards. The universal three questions asked of any fitting routine for a given source model are: 1) is the model in error, 2) are the parameters significant, and 3) are the parameters interpretable. The sECD model meets the third criterion readily, since the sECD represents a relatively focal patch of cortical activity, which makes it a workhorse for source modeling in clinical evaluations for epilepsy surgery. In contrast, models that comprise many overlapping ECDs or more distributed source models are comparatively lacking in this third point, as they can create many alternate interpretations or possibilities for the clinician. Accordingly, the sECD model is the most commonly used and comprehensively validated technique, and gained acceptance as the standard method for clinical MEG performed for presurgical evaluation of epilepsy by the ACMEGS (21).

The test for error of a sECD model answers the first critical question. Crucial to this test is to establish the normal “noise” or “baseline” of the data. The methods of baseline noise measurement can differ across laboratories. In our laboratories, the noise calculation is achieved using the variance and cross-covariance of each channel of data during a baseline period before the spike. The residual is then normalized by this baseline to calculate “goodness of fit” (GOF), which is a test for error that is accomplished through several means and often reported as “normalized variance not explained.” The goodness of fit of greater than 70% is a frequently used parameter of acceptance (5). An alternative, and probably better measure of error, is the “chi-square” test which sums the normalized squared error into a single chi-square statistic (28). If the resultant statistic is too large relative to the number of channels, also known as the degrees of freedom, the model is considered in error and rejected.

If the model is not rejected due to error, the second question is whether or not it is “significant.” Our institutional preference is to confirm the confidence volume (CV), or volume of error, of the dipole localization (29). The CV is the region that encompasses the uncertainty of dipole location due to noise that was established at baseline. If the SNR is low, either because the source is weak or deeply located, the noise would dominate the location estimate resulting in a large CV, which would reject the model as “not significant.” There can also be unacceptable CV's at higher SNR, if the selected region of interest from the sensor array is too small, or if the sECD model is too close to the edge of the sensor array. Therefore, a small CV indicates that 1) the SNR of the ECD is adequate, 2) enough sensors were used for source modeling, and 3) the model was not too close to the edge of the recording array.

It must again be emphasized that a source model must pass both the initial test for error (chi-square; GOF), and subsequent one for significance (CV; SNR). Once accomplished, a sECD can be interpreted as a model of abnormal epileptic activity. The sECD models shown in this paper were accepted based upon the following fit parameters: “reduced chi-square” less than 2 (i.e., the chi-square statistic is not greater than twice the number of channels), GOF ≥ 80% (defined as 100% minus the normalized variance not explained), CV less than 1,000 mm^3^, and dipole strength between 100 and 500 nAm.

### Clinical Integration of Dipoles (MSI) and Conventional EEG

It is accepted that MEG and EEG are complementary, each providing a different perspective. More importantly, in MEG performed for epilepsy surgery evaluation, simultaneous MEG and EEG recordings are recommended as a clinical standard (21, 30, 31). We would further endorse this to the extent that simultaneous MEG and EEG recordings are, in fact, required for MEG performed for epilepsy surgery. The importance of EEG recording in MSI are to 1) exclude known benign-epileptiform EEG variants that can present in MEG, 2) evaluate significance of dipoles localized to regions associated with benign MEG-unique variants, 3) increase detection of MEG waveforms with low SNR, 4) determine source localization credibility, and 5) distinguish EEG unique spike types.

It has been shown that benign epileptiform EEG variants such as sleep transients can be presented in MEG, and simultaneous EEG recording can prevent these waveforms from being modeled and erroneous reported as pathologic. Benign MEG-unique variants were also briefly alluded to during the discussion of model worthiness and will continued to be discussed in relation to illustrated anatomical contexts. In summary, this is a term describing MEG sharps or spikes without epileptiform EEG correlate that are localized to specific cortical regions, and are unlikely to have pathologic significance in most patients (27, 30). However, if these MEG spikes were shown to have epileptiform EEG correlate, these spikes would be considered pathologic and their models would be reported. Without concurrent EEG recording, the interpretation of these MEG dipoles would be limited. From these standpoints, EEG recording is necessary for accurate dipole interpretation for epilepsy surgery.

EEG recording also aids the detection of less visible MEG signals. MEG is attuned to sources localized to deep sulci, fissures, and cortical planes with tangential fields, but has less detection capability in sources that are radially oriented. However, predominant radial sources can still have some smaller tangential component, and EEG can be utilized as a detection tool. In this context, the MEG signals from these sources can be detected through averaging using the EEG as the trigger (Figure 3B).

**Figure 5:**
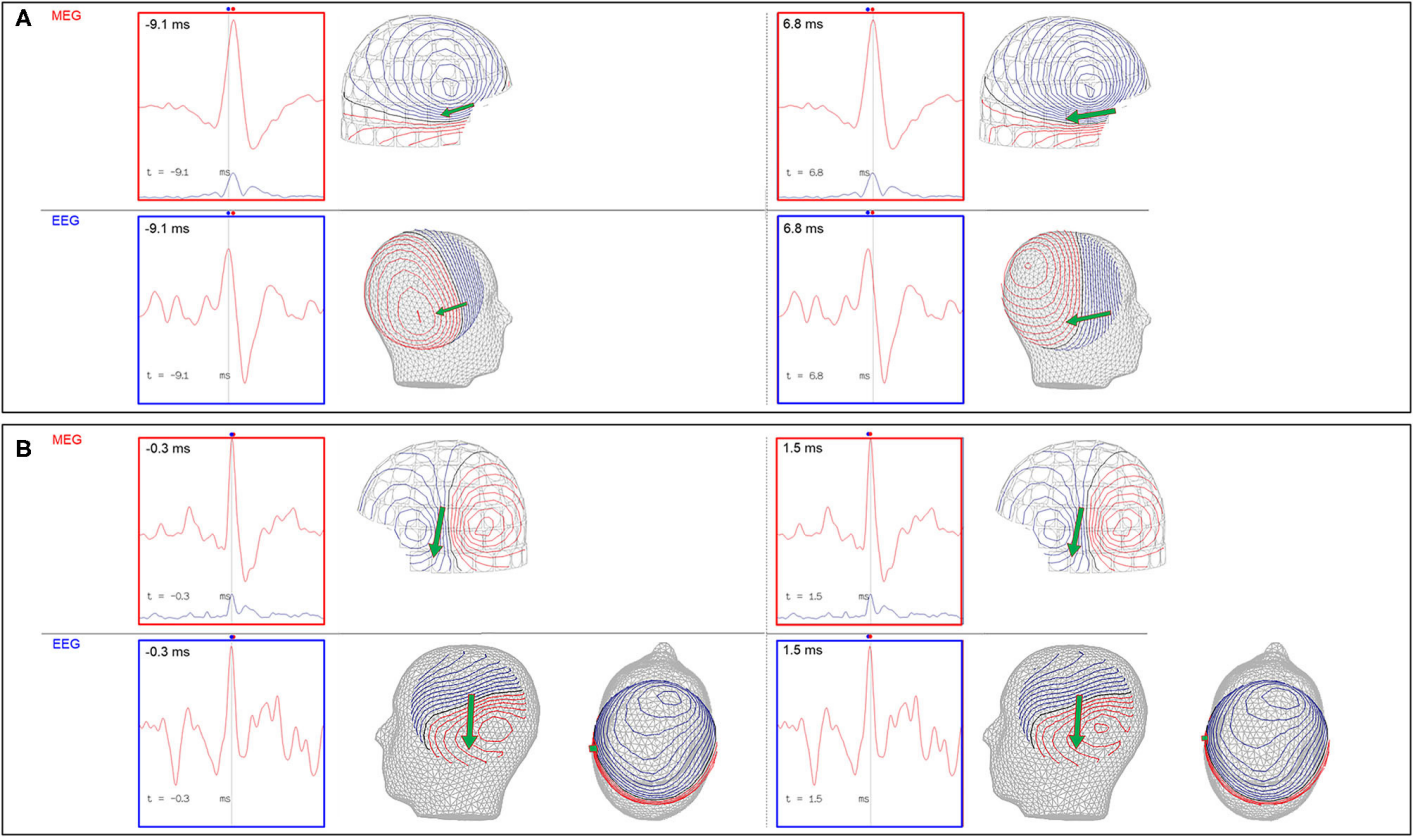
Importance of MEG-EEG integrative analysis (red dots: time of MEG peaks; blue dots: time of EEG peaks; green arrow: MEG source localization). **(A)** Right temporal MEG spike peak lags that of the EEG (15.9 ms). Although the dipoles are congruent, the earlier EEG spike with an anterior field pattern raises the possibility of a preceding anterior source. **(B)** Left temporal MEG spike without a significant MEG-EEG peak latency difference. Although this patient's 22-channel scalp EEG tracing showed bifrontal maximal negativity, this EEG field pattern can be explained by both the MEG and EEG dipole sources.

The EEG is also used to support an MSI result. There are two important points to consider for comparative MEG and EEG analysis, which are 1) MEG-EEG peak latency differences, and 2) congruent source orientation. The latencies between MEG and EEG peaks are useful in determining whether MSI is likely to represent the source or propagation pattern. A MEG peak that is significantly delayed compared to the EEG peak may suggest that the MEG peak is potentially a propagated activity. An MEG-EEG peak latency difference of greater than 10 ms is considered significant in our laboratories. However, it should also be noted that a study of frontotemporal lobe spikes demonstrated that the observed propagation of peak activity can be more rapid in EEG, whereas propagation of the MEG peak had a velocity similar to the intracranial EEG recording (32). The congruence of source orientation has to also be considered, and this can be preliminarily determined from the EEG field map (Figure 5).

MEG and EEG unique discharges can occur across studies (33, 34). Such EEG waveforms should always be averaged to assess underlying MEG signals. If no MEG correlate is found, the presence of these EEG-unique discharges must be noted, as they can represent different sources. Furthermore, it is clinically useful and important to confirm that typical epileptiform EEG discharges of concern were captured during the MEG study.

Comprehensive MEG interpretation must take into consideration the EEG correlate, particularly if the MEG peaks are significantly delayed compared to EEG, if the MEG and EEG field patterns are discordant, and when there are EEG-unique sources without an MEG correlate even after averaging. Under these conditions, the source representation through MSI would be incomplete, and EEG source imaging (ESI) would be beneficial (30). Although the current version of the ACEMGS clinical practice guideline (21) does not indicate an inclusion of ESI with MSI as a standard procedure, this may change in the next iteration (35).

### Reflection on Integrated Use of MSI and ESI

ESI is a source localization technique using the EEG signal, which adds an additional dimension to results obtained from MSI. Specifically, it provides confirmation of MEG source configuration, adds the source's radial component, assists in evaluation of sources where the EEG significantly precedes MEG and where MEG may represent a propagated activity, and localizes EEG-unique radial sources. Some physiologic limiting factors exist with ESI, since an EEG spike require a larger cortical activation area (36), and the localization results are typically deeper than MEG (30). ESI also requires an increased number of electrodes than that typically used to increase source localization accuracy, such as at least 32 channels (37). From forward modeling using a human skull phantom and comparing 122 channel MEG to 64 channel EEG recording, the averaged localization error from EEG (BEM: 7.63 mm; spherical 8 mm) is greater than MEG (BEM 3.4 mm; spherical 4.14 mm) (38).

Despite some localizing limitations, there is utility to ESI given that it complements MSI, and EEG data is readily available. However, implementation of ESI is limited by the complexity of its volume conductor model (36, 39). In contrast to a magnetic field, electrical activity traversing from the cortex to the skull passes through spaces with different conductivity values. Because of this, a conductor model for ESI is more complicated, and typically includes at least 3 layers comprising of brain, skull, and skin. There are models with even a greater number of compartments, and localization depends on conductivity values and ratios (17, 18).

The strengths and weaknesses of ESI in clinical practice has been reviewed (36, 39). Aside from certain conditions that were described here, the authors have noted the ability of ESI to assist localization of temporal and basal sources, whereas it has limitations in frontal lobe epilepsy. These two reviews supplement the original article on the combination of MEG and EEG source modeling (30). It must be emphasized that the added benefit of ESI should always be considered in patients possessing EEG spikes that precede MEG spikes and EEG-unique spike types. However, as there is no current national or international practice guideline for ESI (35), its usage remains complementary to MSI (5) and conventional EEG analysis.

### Dipole Clusters: Definition, Types, Clinical Interpretation, and Significance

**Figure 6:**
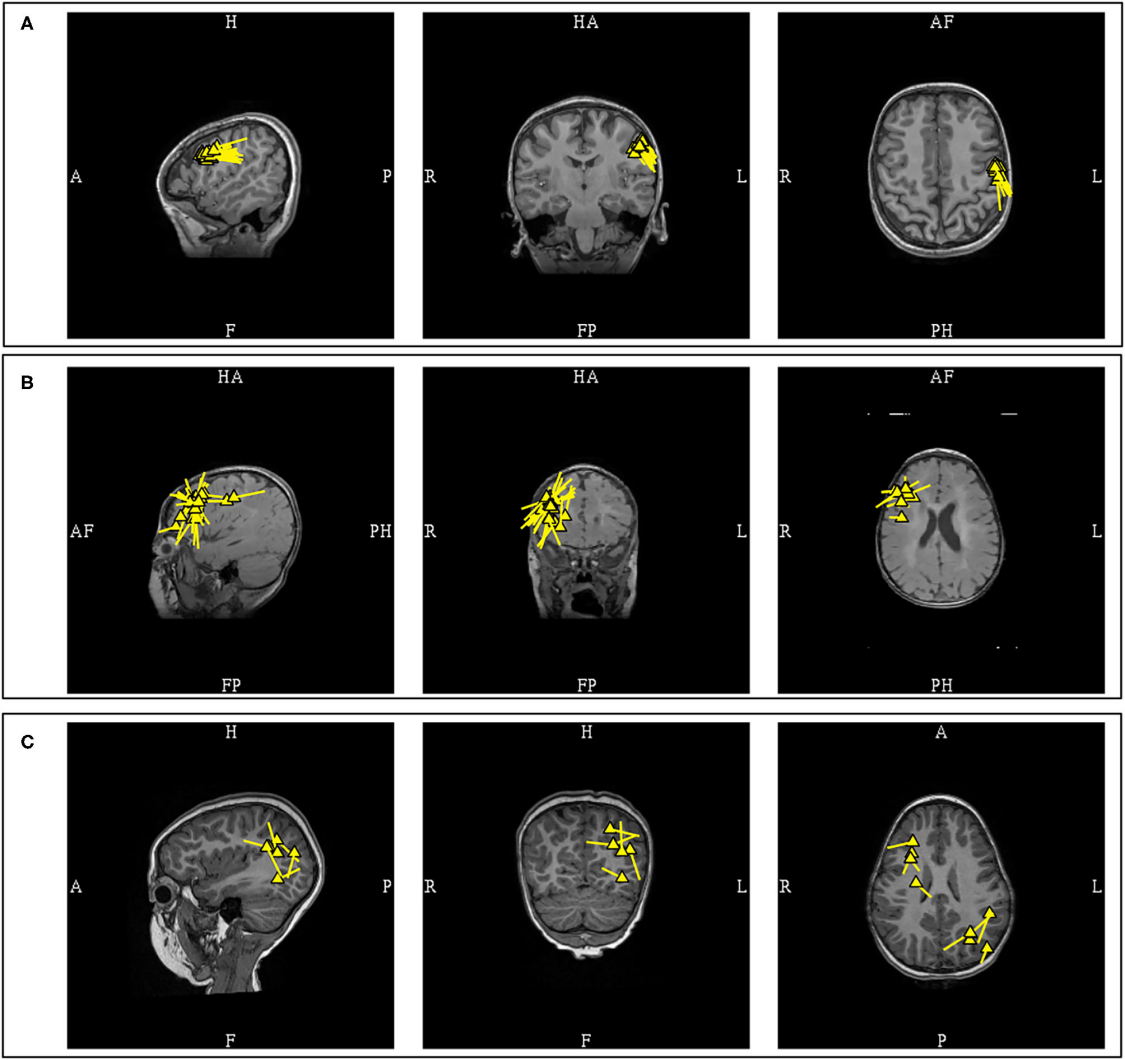
MEG-MRI coregistration summary demonstrating varied cluster patterns. **(A)** Monofocal tight cluster with uniform vector orientation localized to the left pre-central region, with dipoles posteriorly oriented. **(B)** Monofocal loose cluster with variable vector orientation localized to the right dorsolateral frontal region, with additional posterior scattered dipoles. **(C)** Multifocal and scattered dipoles over the right fontal and left parieto-occipital regions.

A “cluster” is a frequently used term in sECD modeling to describe a pattern of distribution or grouping of individual spike dipoles that are localized closely together within a volume. Although this is a commonly used term in clinical MEG, it currently does not have a standard definition. There are two variables that would define a cluster, first is the number of dipoles, and second is the volume which it occupies. Different author groups have proposed and used varying numbers, but the number of at least five dipoles used in some publications (11, 40) is probably conservative and falls in line with the ACMEGS recommendation that a minimum number of 5 model worthy MEG epileptiform discharges should be present in a study to be clinically sufficient for interpretation (5). The volume of involvement also differs in the literature, as different criteria reflecting either anatomical regions (11) or mathematical measurements (41) have been used. Since there is no standard definition, epileptologists typically refer to dipoles that are localized closely together as “a cluster,” and those that are more loosely dispersed as “a scatter” or simply scattered. It is likely that this definition gap will be closed in the future as the work on harmonizing clinical MEG practice internationally is advancing (35) (Figure 6).

Dipole clusters can provide insight into the nature of underlying pathology, and guide subsequent surgical planning. Three factors that should be considered when approaching dipole clusters are 1) the number of clusters and their distribution, 2) density of dipoles within a given cluster and their orientation uniformity, and 3) presence of a radiologic correlation. Patients with a single dipole cluster (42–45) and those whose dipoles are confined to the same lobe (46) have been found to have more favorable post-operative outcomes. This is additionally supported by the finding that a monofocal cluster is more likely to overlap with the ictal onset zone, while multifocal clusters may reflect a widespread epileptic network (42). Lower dipole density may suggest a regional hypothesis, as evidenced by the finding that the cluster in Type 1 focal cortical dysplasia, commonly associated with lobar atrophy (47, 48), are looser or scattered in comparison to Type 2 and 3 (40), and those with dense clusters have better post-operative seizure freedom outcome (49). Dysplastic tissues are associated with less spike-variance (50), and inconsistent dipole orientations can signify underlying widespread epileptic network (51). These understandings are further substantiated by the recent study which associated monofocal clusters and dense dipole clusters with uniform orientation with a better operative outcome (11). A very recent study in MRI-negative pediatric patients, using inter-dipole distance of 15 mm to define “clusterness,” also showed that dipoles that clustered were closer to seizure onset zone (16.2 mm) than those that were scattered (30.4 mm) (52).

Radiologic correlation is also an important factor in the integration of dipole clusters. The presence of a contributory lesion close to a MEG cluster would supports its epileptogenicity (53). The role of MEG also extends to the identification of a probable contributory lesion in MRIs with multiple lesions, or in lesions of indeterminate significance (54). In MRI-negative epilepsy, the presence of an MEG cluster should prompt a radiologic review, especially since MRI abnormalities may not be readily appreciated from initial interpretations (55–57). It should be noted that an MEG can identify epileptogenic lesions that remained unidentified under conventional three Tesla MRI (57, 58). The size of the dipole cluster compared to the size of the imaged lesion is also variable. In a study of focal cortical dysplasia with 1.5T MRI that used a correlation coefficient of greater than 98% and a CV limit of 5 cm^3^ as an acceptance parameter, more than half of the dipole clusters were larger than the lesion (*n* = 11/21); 33% were similar to the lesion (*n* = 7/21); and 14% were smaller than the lesion (*n* = 3/21) (41). A non-Type 2 cortical dysplasia was also more likely to have a cluster larger than the MRI lesion as compared to Type 2 (70 vs. 36%) (41).

Aside from the fact that underlying pathology affects the size of a dipole cluster, evidence also exists that modeling parameters can also affect dipole density. SNR has an inverse relationship with CV (59), and it has been shown that a cluster would become more dispersed with incremental noise introduction (60). As such, localization of dipoles with large CV or modeling sources with low SNR can result in loose clusters or scattered dipoles. Clinical MEG practitioners must be aware of these issues and their potentially misleading effect on the incorporation of the MEG results in an implantation scheme and resection plan.

The finding of a monofocal and dense MEG dipole cluster with uniform orientation and a corresponding MRI lesion would nicely satisfy a restricted focal hypothesis. In practice, supplemental electrocorticography may still be required to establish the full extent of the irritative zone (61). The presence of scattered dipoles, multifocal clusters, or loose clusters with variable dipole orientation and the absence of a corresponding lesion, are all factors that support a regional or network hypothesis. This is especially when these loose clusters or scattered dipoles are observed under fit parameters that utilized small CV limits or higher SNR values. However, even in these cases MEG remains useful as it can provide an approximate anatomical location of the involved regions from which epileptologists can formulate an epileptic network prior to invasive recording.

### ECD Modeling of Ictal Onset

Ictal events can occur during MEG studies, and a recent review reported seizures during MEG in 7–24% of patients (62). Ictal MEG provides useful source information at the time of seizure onset, in addition to that of interictal spikes. However, in addition to the modeling challenges and necessary cautions, limitations on ictal data interpretation can occur if the signal is of low amplitude or there is excessive myogenic artifact.

Currently, there is no consensus on how to model the MEG of ictal onset. We have identified 14 articles on ictal MEG to compare their approaches, including modeling methods and localization findings. ECD modeling was used utilized in 8 publications (63–70), distributed source modeling in 4 articles (68–72), beamformers analysis in 3 studies (69, 72, 73), multiple signal classification (MUSIC) in 1 report (69), and maximum entropy of mean (MEM) in 1 study (74). Some authors used more than one modeling method (68, 69, 72). In one report the source localization method could not be determined (75). From these data, we found that ECD modeling is still a widely used method for MEG ictal onset localization.

Despite this, we have also found that ECD modeling methodologies also differ among the groups. There appear to be two basic approaches: 1) modeling individual early ictal waveforms (5 groups) (63–67), and 2) modeling averaged early discharges (2 groups) (68, 70). Modeling methods also varied between single dipole fit, sequential dipole fit, or multiple dipoles fitting. One study that utilized ECD modeling did not detail its procedure (69). Although ECD modeling techniques showed differences across the publications, the resultant outcome of all appears to be favorable. One older study advocated that an ictal MEG as at least equivalent to invasive EEG in 5 out of 6 patients (64).

**Figure 7:**
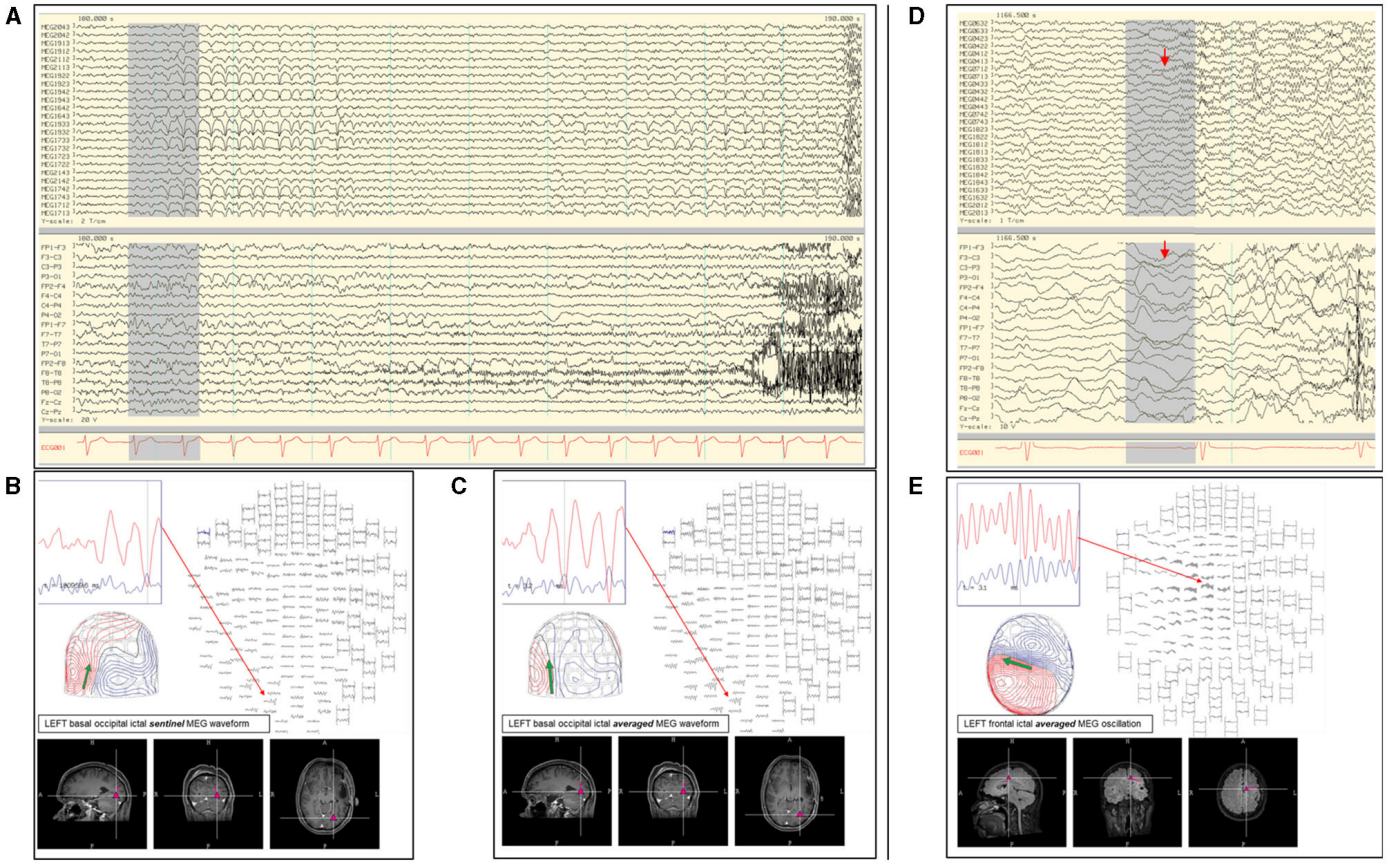
**(A)** Ictal MEG-EEG recording of a patient showing MEG onset at the left occipital channels (MEG1922; MEG1942; MEG1933; MEG1733) with corresponding left parieto-occipital EEG changes. Single ECD analysis of **(B)** sentinel MEG sharp wave and **(C)** averaged MEG rhythmic activity showed consistent localization at the left inferior occipital lobe. **(D)** Ictal MEG of a patient with broad left hemispheric encephalomalacia who lacked interictal MEG and EEG discharges, with ictal EEG onset showed oscillating activity over the left mid-parasagittal region and MEG channels showing a corresponding sustained faster frequency (MEG0712). **(E)** Early oscillating signals during the initial 120 ms were averaged showing a dipolar field suitable for modeling, which was localized to the left frontal region and used to guide electrocorticography.

In our practice, we have adopted both the modeling of individual and averaged early ictal waveforms (Figures 7A–C). The isofield of the sentinel waveform is analyzed to determine its stability prior to modeling. In cases with multiple repetitive spiking, the source of each would be analyzed individually and chronologically. However, the modeling of sentinel spikes does have limitations, since ictal onset pattern may be low signal-to-noise fast activity, while later more visible ictal spikes are in fact propagated activities (21). Despite its high spatial resolution, sECD localization of the ictal origin depends on modeling of the earliest recognizable ictal MEG activity and not subsequent propagated activity (76). This is a factor to consider always when modeling ictal discharges, especially those from sources in the interhemispheric fissures that are prone to fast contralateral propagation. These propagated signals may be more visible which can result in false lateralization. Averaging of early ictal waveforms or oscillations can be a useful method for modeling of seizure onset, as it increases SNR and reduces dipole variance (Figures 7D,E). Bandpass filtering of 3–15 Hz for temporal seizures and 3–25 Hz for extratemporal seizures can be used to improve MEG and EEG ictal data with excessive noise or artifacts that significantly impair signal selection and source analysis.

Sometimes seizures cannot be analyzed by an ECD. In a study of 44 patients with ictal MEG, sECD modeling was successful in at least one seizure in 66% of the patients, but there were other seizures that could not be modeled in this way and required extended source models (63). This group suggested that a resection area guided by MNE has a stronger correlation with seizure freedom, and they advocated using extended-source localization as a primary method of ictal MEG analysis. In contrast, another study that implemented multiple methods [sECD, sLORETA, MUSIC, and SAM(g2)] reported no difference between localizations using sECD and extended source models (69). We view that magnetoencephalographers should also learn to use extended source modeling techniques, especially since ECD modeling of ictal onset may be unsuccessful or the result questionable. When in doubt, a magnetoencephalogrpher should compare the ECD model of ictal onset with the result obtained from another modeling method.

## Clinical Interpretation of Dipoles Based on Anatomical Location

### Temporal Lobe Dipoles

The temporal lobe is a complex anatomical structure, with many surfaces and varied propagation patterns. Classification of temporal dipoles into those of anterior and posterior regions have been suggested (77). These can be further sub-classified into three groups: anterior temporal horizontal (ATH), anterior temporal vertical (ATV), and posterior temporal vertical (PTV), that correlate with temporal tip, anterior superior, and posterior superior temporal planes sources, respectively (77). The orientation of these dipoles are in reference to the cortical anatomy. The ATH and ATV dipoles are associated with anterior temporal sources (mesial, entorhinal, temporal tip), and some ATV can represent a later ATH propagation activity. The PTV dipoles are more commonly associated with lateral neocortical surface, superior temporal plane, temporal base, and structural lesions, but invasive recordings have shown that seizure onsets associated with PTV dipoles can also be unlocalized or mesial in origin. This anterior and posterior classification was later reaffirmed (78).

It was hypothesized that the source origins of ATH and ATV dipoles should lie within the resection boundary of standard temporal lobectomy (77). Surgical outcomes through this approach was later investigated in patients diagnosed with temporal lobe epilepsy (79). Using the central sulcus as the landmark, the patients were classified as anterior temporal group if there were greater than 70% of dipoles localized to the temporal lobe anterior to the central sulcus. In the absence of neocortical lesion, standard anterior temporal lobectomy in anterior temporal MEG group was associated with good outcome (100% Engel 1; *n* = 5/5). In contrast, the outcome in non-anterior MEG group were variable (67% Engel 1; *n* = 4/6), and presence of extratemporal dipoles were noted in some of these patients who continued to have seizures.

**Figure 8:**
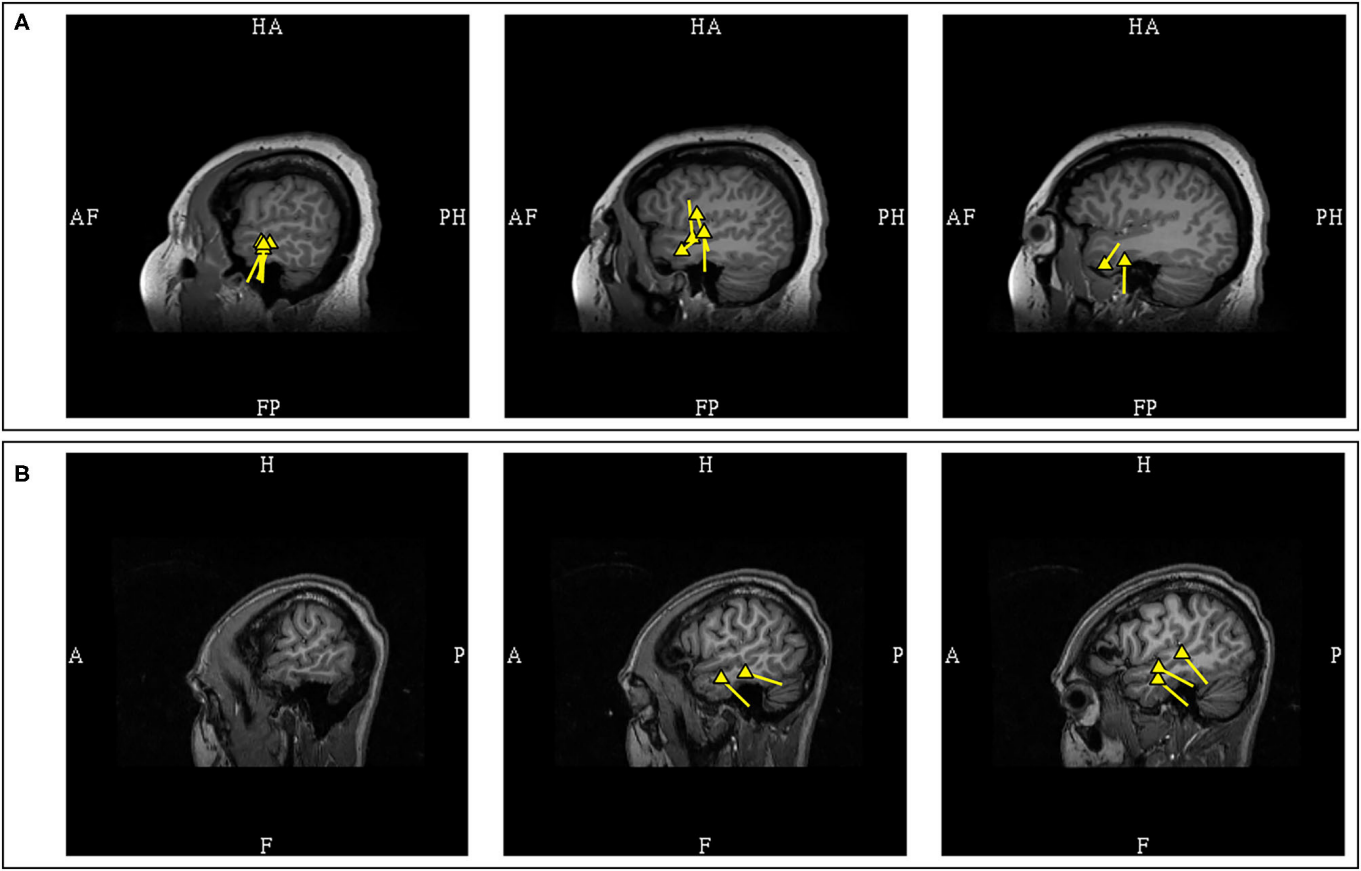
MEG-MRI coregistration summary in two patients with temporal dipoles. **(A)** Frequent anterior temporal dipoles were suggestive of anterior temporal source. Imaging review revealed temporal tip encephalocele. **(B)** Temporal dipoles broadly distributed over anterior and posterior temporal region provides comparatively limited suggestion of seizure onset in the absence of a lateral temporal lesion.

A study in patients with established mesial temporal lobe epilepsy found that the patient's MEG dipoles were localized to the anterior temporal region, without posterior or extra-temporal localization (80). Another study in mesial temporal lobe epilepsy also reported that the dipoles were localized to the anterior temporal lobe, with the majority of the dipoles being horizontal or mixed (81). This study also noted that non-concordant localizations were found with predominantly vertical dipoles suggesting propagated activity, but the presence of posterior temporal dipoles was not mentioned. Presence of temporoparietal MEG propagation in mesial temporal epilepsy has also been associated with continued seizures after epilepsy surgery (82). The initial separation of the temporal MEG dipoles into anterior and posterior divisions is a practical approach with surgical relevance, and the presence of predominantly anterior temporal dipoles is more convincingly suggestive of anterior or mesial temporal sources (Figure 8).

**Figure 9:**
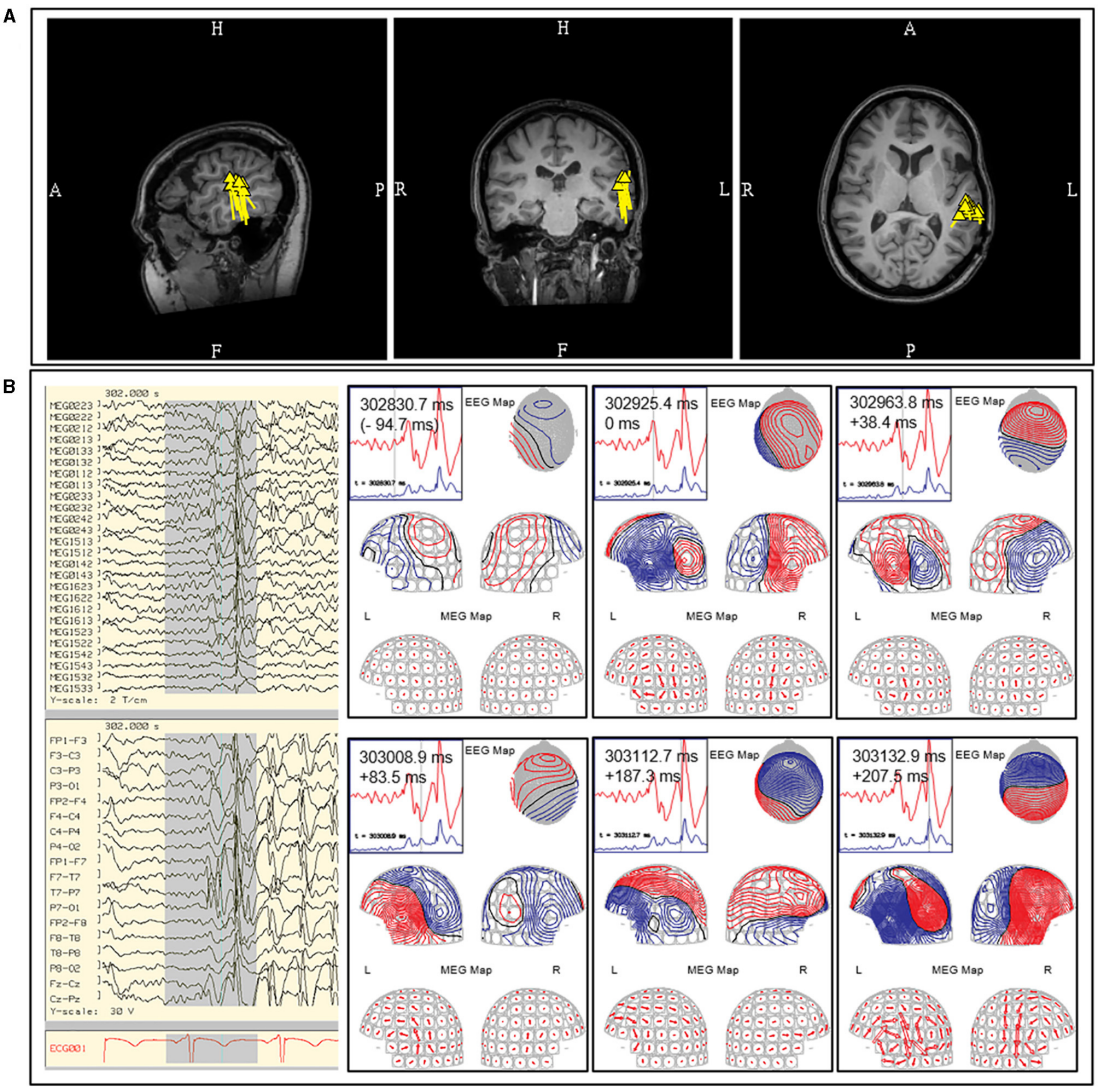
Example of posterior temporal vertical dipoles that represent propagated activity. This MEG was performed in a patient with prior subdural recording which indicated a left frontal onset, but continued to have seizure after resection. The MEG study contained both **(A)** independent focal left posterior temporal discharges that formed a tight and uniform cluster and **(B)** bilateral posterior temporal discharges that were rapidly synchronous (upper middle diagram at 302925.4 ms; initial peak defined as 0 ms; left-right peak difference <10 ms). Data from prior subdural recording and the presence of bilateral synchronous discharges suggest alternate source of origin for this MEG cluster. The MEG current (arrow plot) suggested an underlying left frontotemporal current that cannot be modeled. These findings were described to the referring epileptologist.

Posterior temporal dipoles are comparatively more variable and can be divided into three categories: 1) benign MEG-unique variant, 2) pathologic and lesional, and 3) pathologic but non-lesional. Benign MEG-unique variants can be observed in the posterior temporal region over the peri-Sylvian area, and dipoles localized here are typically benign especially if they are bilateral or have 180 degree opposing orientations (27). Suggestive features of epileptogenicity, aside from EEG correlation, may include unilaterality, uniform orientation, and clinical suspicion. For pathologic PTV dipoles, the presence of a corresponding lateral temporal lesion would suggests that these dipoles are likely from a lateral temporal source (78). However, additional possibilities need to be considered in non-lesional patients as PTV dipoles have also been observed in patients with seizures of mesial temporal (77) and operculo-insular onset (83). Similarly, we have also observed PTV dipoles that are unlikely to originate from lateral temporal lobe in our practice (Figure 9). Subdural EEG recordings in some patients with temporal epilepsy and PTV dipoles also reported widespread seizure onset involving both medial and lateral temporal contacts (78). Clinical context and experience is therefore needed to interpret dipoles localized to the posterior temporal region.

### Frontal Lobe Dipoles

**Figure 10:**
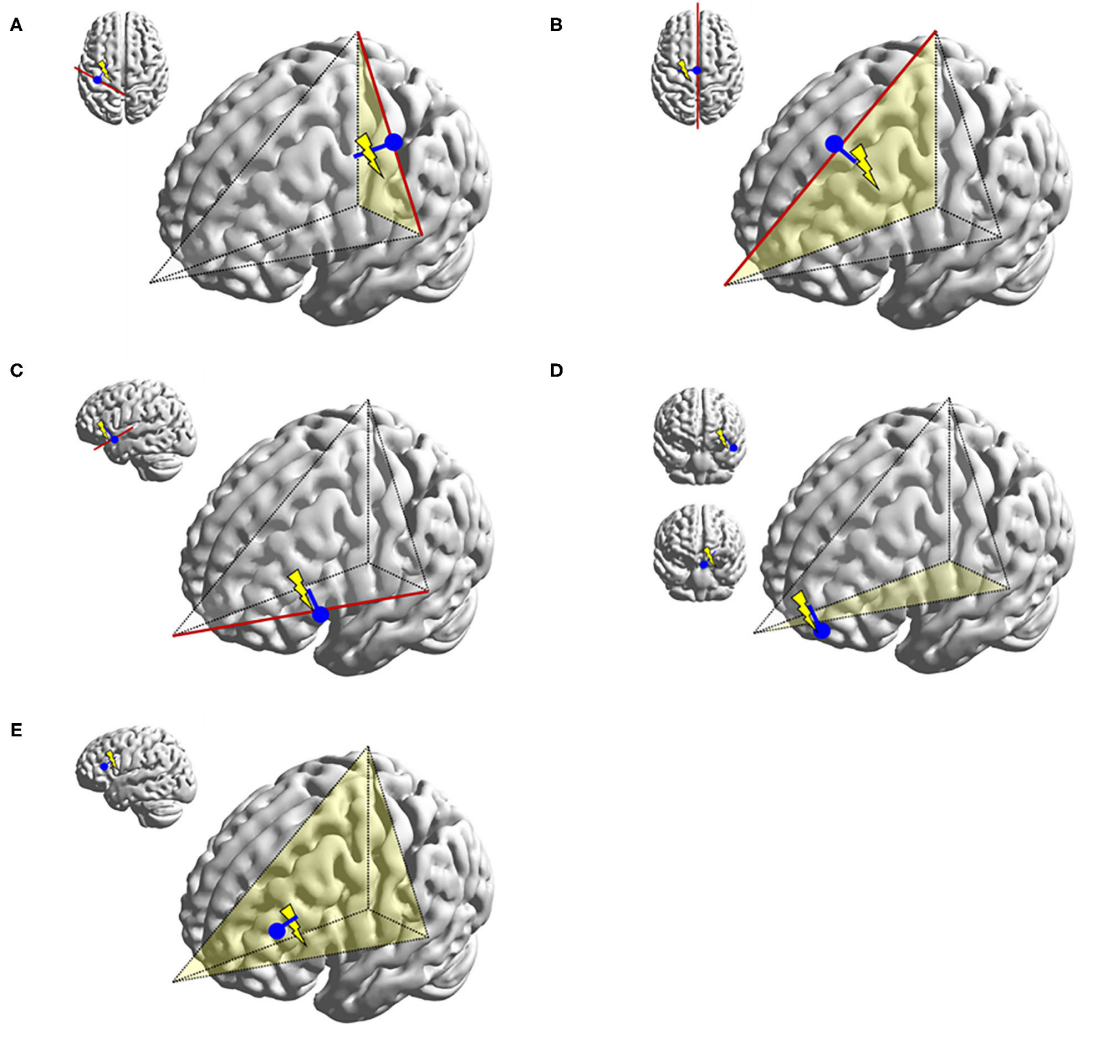
Illustrations representing typical dipoles (blue) in correlation to frontal spike sources (electric symbol). **(A)** Peri-Rolandic frontal sources exhibit dipoles with anterior vector orientation. **(B)** Left medial frontal source exhibit lateral vector orientation to the left. **(C)** Fronto-opercular source exhibit upward vector orientation. **(D)** Orbitofrontal sources exhibit superior vector orientation similar to fronto-opercular source, but somewhat more oblique. Dipole tails of lateral orbitofrontal sources point more medially and those of medial orbitofrontal sources more laterally. **(E)** Lateral frontal dipole is shown here with dipole direction oriented toward the source, but its localization would also be dependent on the dipole cluster topology. This diagram can also be applied to parietal and occipital dipoles.

**Figure 11:**
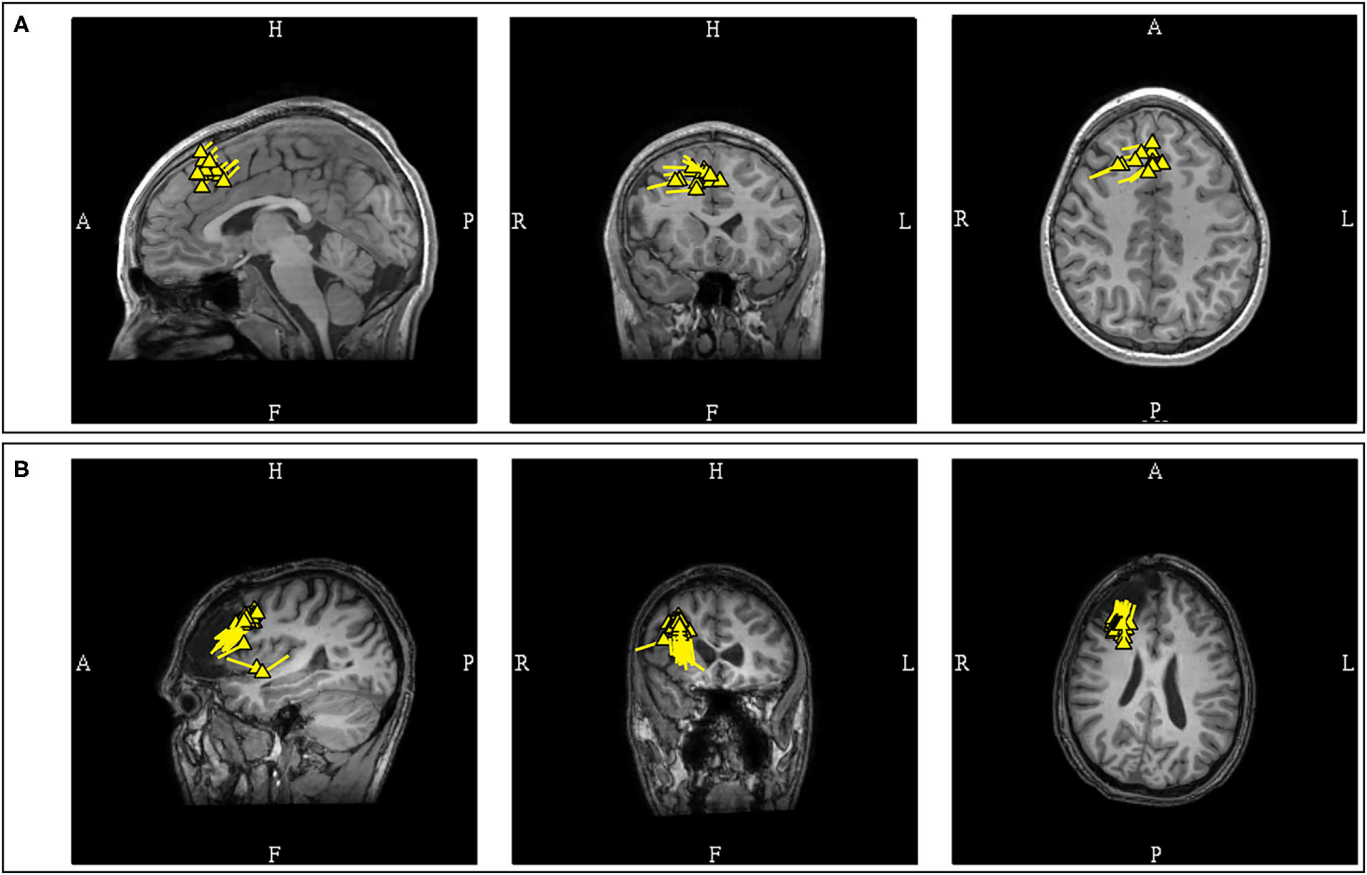
**(A)** MEG-MRI coregistration summary demonstrating predominantly interhemispheric frontal dipoles. The uniform vector orientation toward the right suggests a right hemispheric source lateralization. Intracranial recording confirmed the finding, with seizure freedom for 5-years after right frontal resection. **(B)** This patient developed late seizure recurrence, with tight and uniform dipole cluster at the resection margin. Note the dipole orientation suggestive of positivity at the resection surface as a result of disruption of normal cortical laminar organization.

The frontal lobe is the most frequent location for MEG spikes in extratemporal epilepsies (84, 85), and for this lobe MEG has shown a better yield as a localizing test than EEG (86). Using easily identifiable anatomical fissure boundaries (Sylvian, interhemispheric, and Rolandic fissures), the frontal lobe can be separated into four anatomical surfaces: orbitofrontal (inferior), lateral, medial interhemispheric, and posterior peri-Rolandic (posterior) surfaces. The interhemispheric and peri-Rolandic sources of frontal origins will discussed in the later segment, but dipoles from these sources would typically exhibit orientation toward the frontal lobe (87, 88) (Figures 10, 11A).

Orbitofrontal epilepsy can have variable electrographic and clinical findings, and the literature has demonstrated cases where MEG is a useful localizing tool (86, 89) even when other ancillary studies were negative (90). The data on MEG dipoles from this source is limited, but it is observed that MEG dipoles from this region are typically of basal frontal origin with upward tail orientation. Lateral orbitofrontal dipoles are oriented more medially and medial orbitofrontal dipoles more laterally. However, dipoles localized to the lateral orbitofrontal region should also raise the possibility of anterior mesial temporal sources, given its close proximity and common situations where mesial temporal discharges to propagate to the orbitofrontal cortex (91).

The lateral frontal surface comprises of the majority of the frontal cortical area, and MEG dipoles localizations to this region are variable. The interpretation of MEG dipoles in this location relies on cluster topology. A single, dense, and uniform cluster is exceedingly helpful in defining a focal area of interest in such a large anatomical region (11).

MEG has been shown to improve surgical outcome in frontal lobe epilepsies (44). Case series has shown that 90% of MEG dipoles in frontal lobe epilepsy are localized to within 3 cm of the lesion, but data also suggest that underlying pathology may play a role in the proximity of dipole to lesion (43). However, it must be emphasized that frontal MEG findings can be influenced by the depth of the interhemispheric sources (92), rapid propagation time (93), and the mesial temporal-orbitofrontal connection (91). Consideration of potential propagated activity should be always be factored in the interpretation of MEG results in non-lesional patients.

### Posterior Cortex (Parietal and Occipital Lobes) Dipoles

MEG is also useful in the localization of posterior cortex epilepsies (4, 94), including those patients with electro-radiographic discordance (95) or false-lateralizing EEG findings (96). Posterior cortex sources are less common, and represent only approximately five percent of MEG findings in patients with refractory epilepsies in a large study (85), which limits the available literature. A case series of MEG in posterior cortex epilepsies, using linearly constrained minimum variance (LCMV) and MUSIC algorithms, showed accurate detection of irritative and epileptogenic zones with MEG (97). Negative results tended to be from medial and basal sources, which is similar to a study that reported less MEG sensitivity in other anterior basal regions (98). Localization of these basal sources were still feasible in some patients.

**Figure 12:**
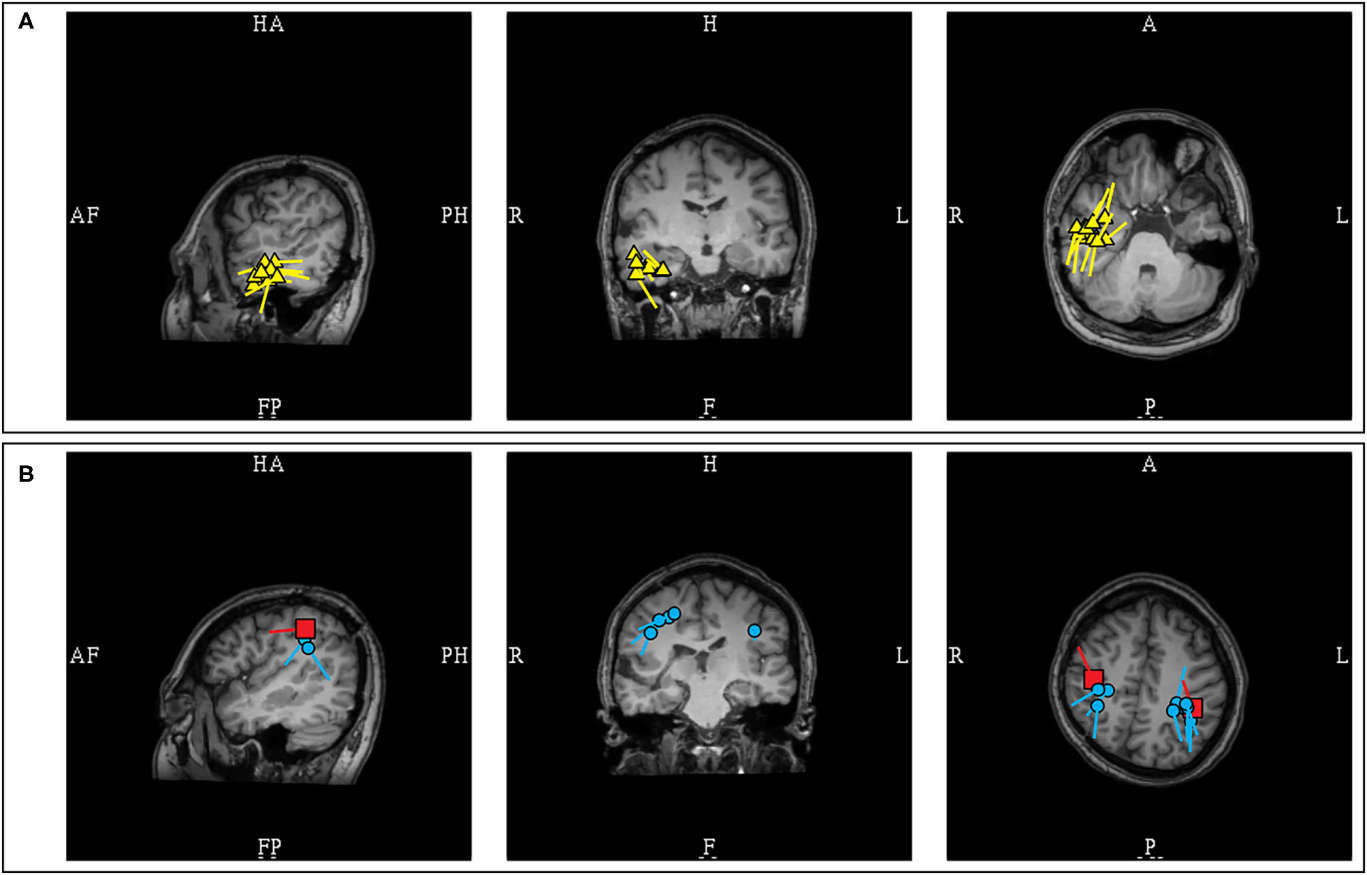
MEG-MRI coregistration summary demonstrating dipoles in a patient with extensive right hemispheric cortical malformation. **(A)** A cluster of likely pathologic right temporal dipoles (yellow) was identified. **(B)** Bilateral parietal dipoles (blue) were also identified in proximity to somatosensory dipoles (red). These dipoles near the somatosensory area, and without an EEG correlate, were considered benign and were not included in final surgical plan.

Careful consideration is required in the analysis of posterior cortex discharges, given that somatosensory, posterior peri-Sylvian, supramarginal (27) and medial occipital (30, 99) cortices are sites of common benign MEG variants. Interpretation of MEG-unique dipoles localized to these regions would require additional ancillary features to determine their significance, and an EEG correlate spike is an important distinguishing feature. Similar to posterior temporal dipoles, bilateral localization, 180 degree opposing orientations, and absence of an EEG correlate are features that would suggest that these discharges are probably benign (27). Clinical context will be required to determine their significance (Figure 12).

Posterior cortex epilepsies have increased representation in pediatric population in the form of benign occipital epilepsy. Although typically self-limited, there are case reports of patients who continued to have intractable epilepsy (100). Given that MEG is typically performed as a part of pre-surgical evaluation, recognizing MEG features of benign focal epileptic syndrome in contrast to a potentially resective etiology is important in pediatric patients. Benign occipital epilepsy of childhood (101), like benign Rolandic epilepsy (87), has sulcal localizations. The dipoles are frequently observed in the parietooccipital and calcarine sulcus, and occasionally in the central sulcus. The variable sulcal locations of MEG dipoles, especially the involvement of the central sulcus, can be an important distinguishing feature of benign epileptic syndromes. However, the study also demonstrated that the dipoles in benign occipital epilepsy can show significant clustering with uniform vectors (a.k.a. dipole orientation), and can be unilateral in some patients (101). This conceivably would lead to its possible presentation as a monofocal sulcal cluster, similar to those commonly associated with lesional, bottom of the sulcus, focal cortical dysplasia (41). This finding should prompt an imaging review, and possibly usage of higher resolution MRI, as such dysplasia can be missed during initial imaging analysis (56, 57, 102). However, the MRI can continue to be negative despite repeated reviews (103), and a surgical recommendation will heavily rely on the epileptologist's clinical assessment. Additionally, MEG can also play a role as a prognostic marker in children with benign occipital epilepsy, as the presence of MEG dipoles outside of these typical sulcal regions have been reported in patient with atypical course and medication resistance (104).

### Interhemispheric Fissures and Major Sulci Dipoles

**Figure 13:**
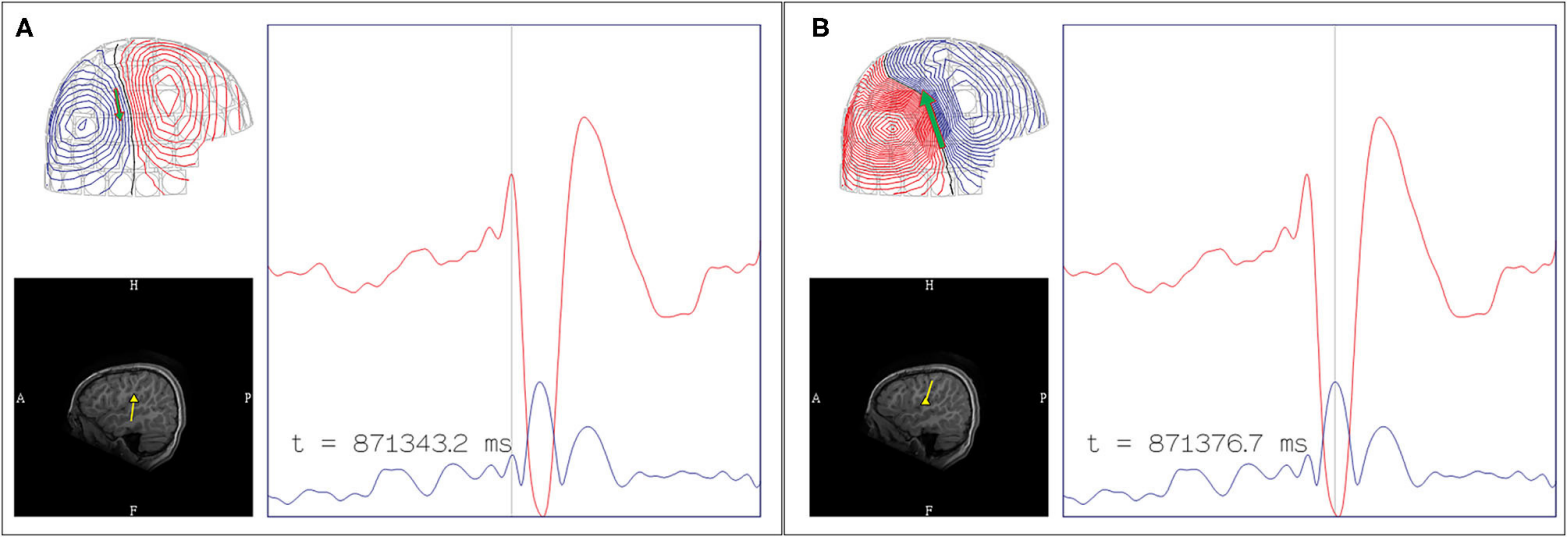
Multiphasic MEG signal with phase-dependent orientation. **(A)** The early peak of lower amplitude was occasionally observed, with dipole oriented inferiorly, likely represents the initial depolarization of deeper laminae. **(B)** The later peak of higher amplitude was a more consistently observed, with dipole oriented superiorly suggestive of frontal operculum localization. Although the area of dipole origin was consistent, the appearance of multiphasic MEG discharges across major sulci can affect the application of orientation-based localization, given that the orientation is dependent of which phase of the discharge was modeled. EEG can assist in determining more pertinent depolarization in these circumstances.

The study of MEG spikes from benign Rolandic epilepsy showed localization to the Rolandic, Sylvian, and interhemispheric fissures and further suggested that the dipoles located in interhemispheric fissures and major sulci had tails that were likely to orient toward the lobe of seizure onset (87). This hypothesis was subsequently investigated in patients with lesional epilepsies (105), which supported that the dipole of MEG spikes would consistently orient toward the lobe of seizure onset when located in the central (100%; 4/4) and sagittal interhemispheric sulci (100%; *n* = 4/4). This is a useful lateralizing feature, but with some reservations, as early MEG activity can be less visible due to lower SNR when compared to propagated activity, and medial frontal sources are known to have rapid contralateral propagation (93), hence modeling of interhemispheric MEG spike peaks may at times represent contralateral propagated activity. Experienced magnetoencephalographers would always ensure that the earliest signals were analyzed, and consider the possibility of contralateral propagated activity if the MEG lateralization is discordant to clinical semiology and other ancillary findings. Another important point to also consider is that a spike can be multiphasic, and the dipole orientation is dependent on the phase which was modeled. Considering this factor, it is a practice standard to routinely show examples MEG spike morphology that were modeled, to model the same phase consistently, and to describe phase-dependent orientation variability, if present (Figure 13).

**Figure 14:**
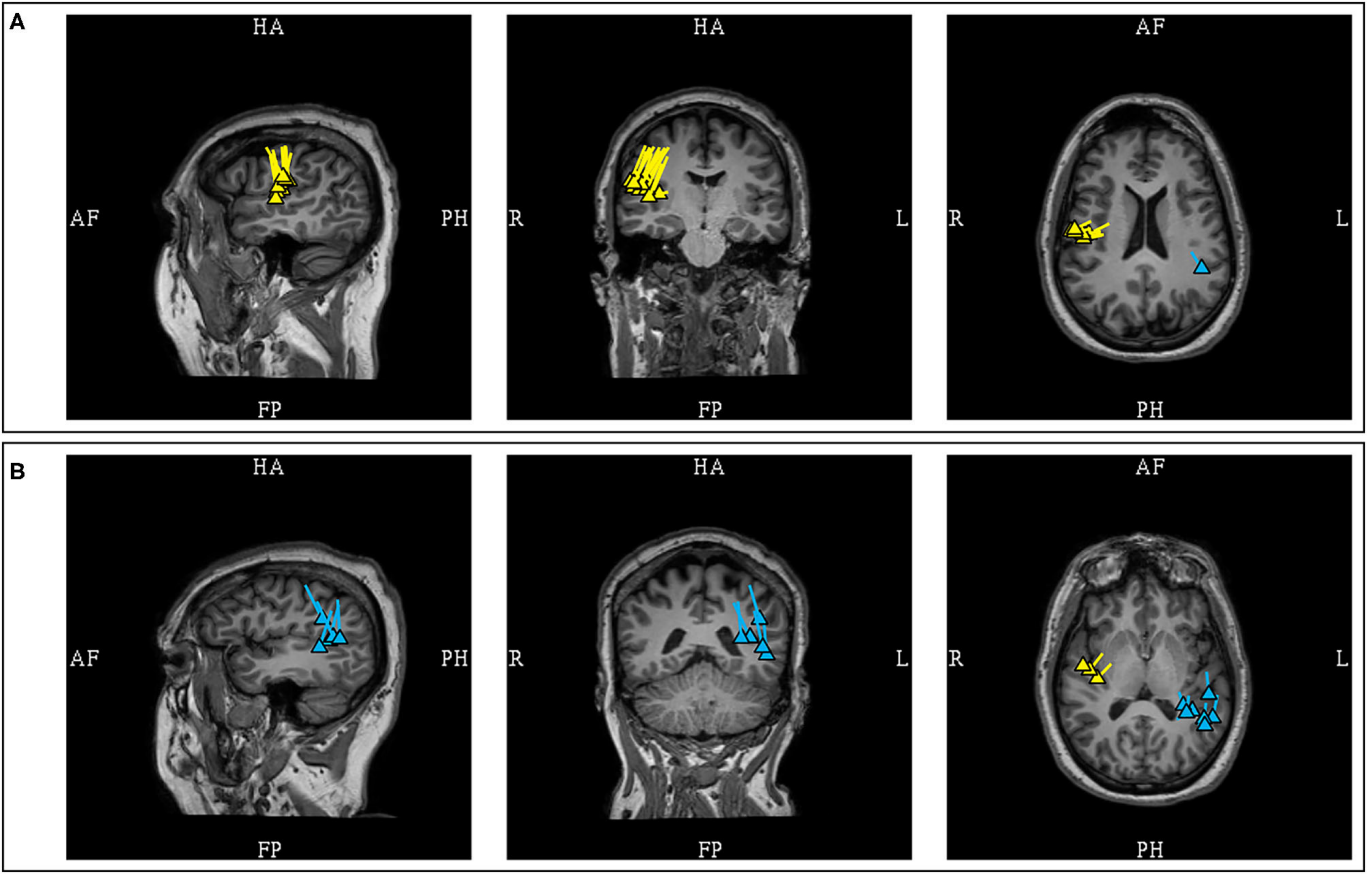
MEG-MRI coregistration summary demonstrating supra-Sylvian dipoles in the same patient. **(A)** The pathologic right mid-supra-Sylvian cluster (yellow) is tight and uniformed, and correlated with a small right fronto-opercular MRI lesion upon imaging repeated review. **(B)** Benign left posterior supra-Sylvian cluster (blue) is loose with fairly uniform orientation. This is a cluster of benign normal variant that has neither EEG correlation nor clinical suggestions, and was not included in the clinical summary report.

The MEG spikes localized to the Sylvian fissures appeared to have variable orientation in respect to the lobe of seizure onset. In a study of Sylvian dipoles which included 8 patients with temporal lobe epilepsy, it was reported that 73% of the MEG dipoles were oriented toward the temporal lobe while 27% were oriented toward to the frontal lobe (105). In contrast, a study of 4 patients with fronto-parietal opercular epilepsy showed that the dipoles were orientated toward the lobe of seizure onset (88). Although these data would suggest that fronto-parietal Sylvian sources are associated with MEG dipoles that are more consistently oriented toward the lobe of origin when compared to temporal sources, the limited number of studied patients and possible orientation variability necessitate that clinical context must be considered in the interpretation of Sylvian dipoles. Furthermore, as previously stated, the posterior peri-Sylvian region is a common location of common benign MEG-unique variants, adding to interpretation complexity (Figure 14).

### Insular/Peri-Insular Dipoles

In clinical practice, MEG has shown utility as a localizing tool in insular epilepsies even in the absence of interictal EEG findings or identifiable structural lesions (83, 106), despite theoretical modeling studies that otherwise suggested an insufficient MEG SNR over this region (107). This importance cannot be overstated, since insular epilepsies have variable clinical presentation and non-specific EEG findings (108). However, utilization of MEG dipoles localized to the insular region can still be a challenge, and at times these are omitted from final surgical resection, given that they can represent propagated activity rather than seizure onset (109).

MEG spikes, depending on source orientation and signal strength, can occur without or with an EEG correlate. The longer history of EEG, and recent recognition of MEG benign variants, support the belief in clinical practice that MEG spikes with an EEG correlate (MEG-EEG spikes) are more likely to be epileptiform. However, due to the lower signal-to-noise properties of electromagnetic signals localized to the insular region, it has been found that dipoles of MEG-unique (a.k.a. “MEG exclusive”) insular discharges can be credibly pathologic and correctly localizing, in contrast to those with an EEG correlate that can often represent a remote propagated activity (110). The analysis of insular MEG sources requires careful consideration of benign vs. pathologic MEG-unique discharges, as well as hypotheses of source of origin based on clinical semiology and known propagation patterns.

It has been shown that anterior insular sources are associated with anterior operculo-insular MEG dipole clusters with anterior vector orientation, typically toward the frontal region (111), and early more anterior source propagation (112). This region is unlikely to be associated with benign MEG-unique variants which are more posterior in location. Hence, it can be stated that the MEG dipoles located in the anterior operculo-insular regions are more likely pathologic and suggestive of an underlying anterior insular source.

**Figure 15:**
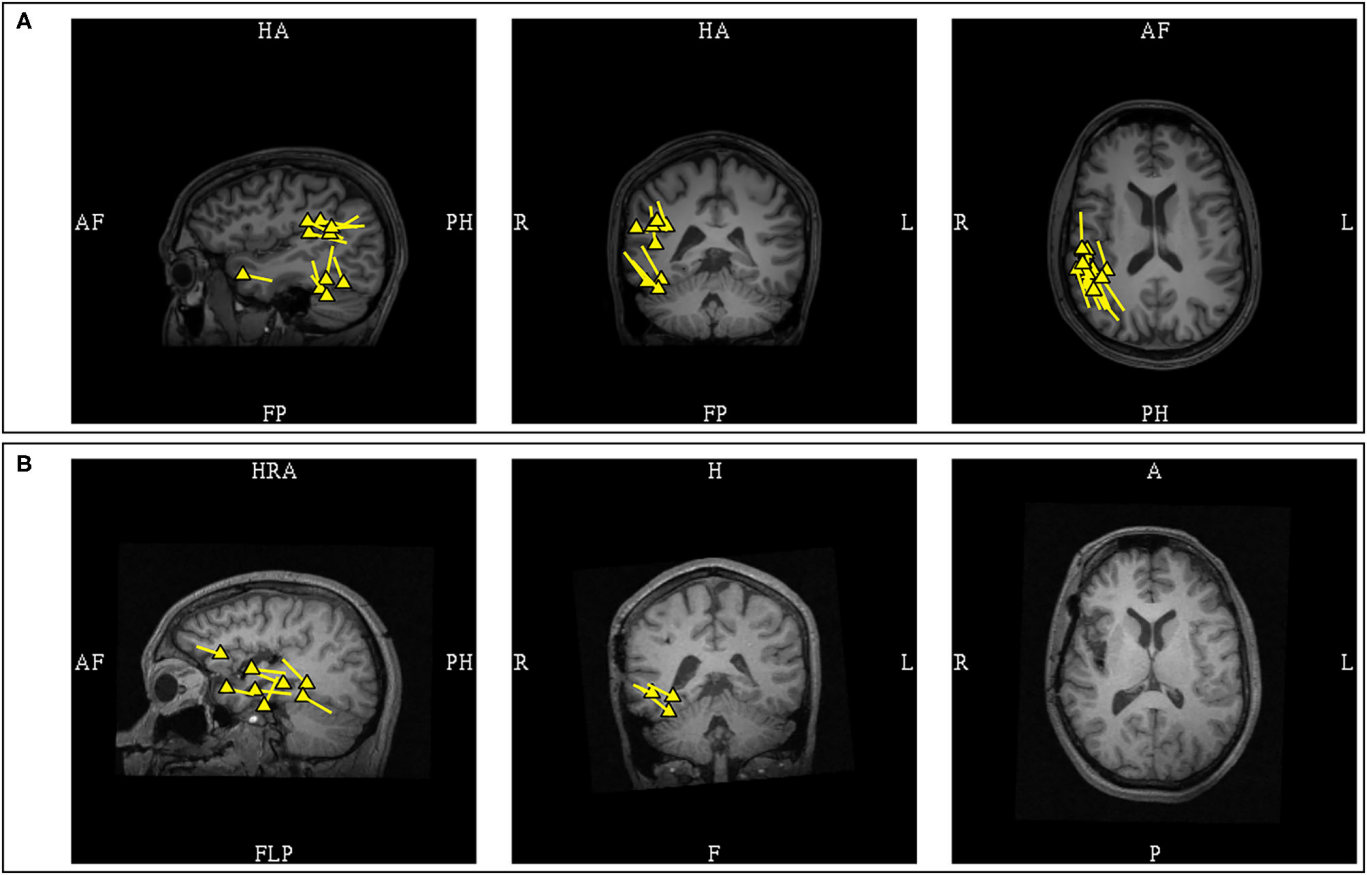
MEG-MRI coregistration summary in a patient with a previous right anterior temporal laser ablation who underwent posterior insular resection. **(A)** Pre-operative MEG showed multifocal clusters over the right supramarginal, posterior basal temporal, and anterior temporal regions. The supramarginal cluster in this patient was pathologic due to presence of an epileptiform correlate. Invasive EEG recording confirmed interictal spiking concordant with MEG. **(B)** Post-operative MEG after the posterior insular resection showed disappearance of the supramarginal cluster, but the temporal dipoles remained.

In contrast, the interpretation of MEG dipoles localized to the posterior operculo-insular region requires additional consideration. Although posterior operculo-insular MEG clusters with a posterior dipole orientation can be associated with a posterior insular source (111), the increased possibility of benign MEG variants from this region necessitates a more cautious interpretation, particularly if these discharges are MEG-unique. Sometimes evidence of an EEG correlation can only be found by averaging triggered off MEG spike peaks. However, pathologic insular MEG dipoles can still lack an EEG correlate even after averaging (106). Clinical context is required to determine the significance of such MEG spikes. Unilaterality and uniform dipole orientation would provide additional support that these discharges are pathologic. One should also note that dipoles associated with insular sources can appear dispersed (111), and posterior insular sources can exhibit early propagation to comparatively remote posterior parietal regions (112) (Figure 15).

### Dipoles in Post-operative Recurrence and Changed Anatomy

MEG should always be performed in post-operative patients with seizure recurrence who consider a reoperation (9, 113). As the MEG signals are not influenced by the skull breech and changed anatomy, unlike EEG, source localization by MEG is superior. Improved seizure outcome has been observed in patients who underwent re-operation when MEG is utilized (114). In this specific population, it has been reported that more than half of the patients have at least one dipole cluster at the surgical margin (115). The presence of these dipoles can indicate a possible ictal onset zone and is particularly useful in patients with a broad resection cavity. However, the possibility also exists that dipoles at resection margins are a result of the resection itself, while the pathology is elsewhere. This is exemplified by the report of a patient with early post-operative seizure recurrence, whose MEG showed both a peri-resectional cluster and another remote cluster at a distant cortical abnormality (56). Accurate assessment of the significance of MEG dipoles near a surgical site is reliant on a variety of factors including prior pathology, character of the resection, changes in seizure semiology, and the timing of seizure recurrence. Dipoles located near the resection cavity in patients with late seizure recurrence are more likely associated with the ictal origin, whereas additional distant foci must be considered in early recurrence (116). Nonetheless, it has been shown that more than half of patients with early seizure recurrence have clinically relevant spikes modellable by dipoles at the resection margin (9), and most reoperations are focused at the prior resection margin (115).

There is also a suggestion that MEG spike dipoles following extratemporal resections may be more localizing (117). A study of recurrent epilepsy after frontal and temporal lobectomy demonstrated that frontal dipoles were more closely localized to the post-operative margin (117). In post-temporal lobectomy, although MEG identified activity in remnant mesial temporal structures that led to successful re-operation, the majority of patients had dipoles localized over the lateral or basal temporal regions further away from the resection margin. The localizing value of these comparatively distant dipoles were not further explored.

It is notable that the tail of MEG dipoles, that typically orients toward the activated cortex, does so under the condition of normal cortical laminar organization. Removal of the superficial cortex can result in dipole orientation changes, particularly if the underside of nearby cortex is exposed in the process. In such cases, residual dipoles may have an opposite orientation (Figure 11B). This finding is similar to positive EEG spikes that similarly can occur as a result of prior surgical procedures or trauma (118, 119). In neocortical epilepsies where dipole orientation can serve as a guide toward the epileptogenic cortex, such situations are noteworthy.

## Critical Importance of MEG Reporting and Proper Communication with Referring Physicians

As referring physicians may not be familiar with MEG techniques and results, MEG reporting and communication are practical issues of critical importance. A clinical MEG report should be ACMEGS CPG-compliant (13), complete, concise, and appropriately structured. Since a simultaneous EEG recording is a required component of every clinical MEG study for epilepsy, its absence or deviation from the 10–20 system must be acknowledged and explained. The MEG information should describe the morphology, location, and frequency of detected MEG discharges, and their localization results. The MEG findings must also be compared with their EEG correlates, and their mutual dynamics explored to the best possible degree. EEG unique discharges must be noted, as this can represent alternate sources not identified by MEG. The impression and conclusion should be concise, providing an anatomical localization summary, pattern of distribution, and accurate representative population frequency (120). Finally, the report must correlate MEG-EEG findings to the clinical context, considering both semiology and radiographic findings into its interpretation. To ensure that all relevant clinical priors are considered during the data acquisition and study interpretation, a channel of communication between clinical magnetoencephalographers and referring epileptologists should be present and maintained until the final delivery of the report. The MEG report must answer the questions from the referring physician, and overall constructed for optimal incorporation to future surgical planning.

## Conclusion

This article is written to serve as a practical introduction to clinical MEG interpretation in epilepsy. It reflects the variability in the strength and degree of evidence for different practically relevant aspects of clinical MEG practice. Naturally, unusual circumstances outside of what have been discussed here can and will occur in the course of one's daily practice. To achieve a comprehensive understanding of MEG, a practitioner must gain procedural experience through extensive clinical use and knowledge through continued literature review. We hope that the information contained in this article has achieved its goal of increasing the understanding of clinical MEG localization and interpretation, elevating the level of clinical MEG service, and improving the surgical outcome of our patients with epilepsy. Ultimately, we hope our effort synergizes with others in the epilepsy and clinical MEG communities to promote clinically indicated but greatly underutilized surgical treatments for patients with DRE.

## Author Contributions

CL performed primary research and authored this research. JE incorporated expert experience, primary editing, and ensured accurate application of concepts. JM contributed to source modeling concepts and fundamentals of model acceptance. AB incorporated expert experience, secondary editing, and constructed the literature framework. AS provided institutional findings and data analysis. GV provided institutional findings and data analysis. MF provided the concepts and led this research. All authors contributed to manuscript revision, read, and approved the submitted version.

## Funding

Research was supported to JM by the National Institute of Biomedical Imaging and Bioengineering of the National Institutes of Health under award numbers R01EB026299 and U01EB023820.

## Author Disclaimer

The content is solely the responsibility of the authors and does not necessarily represent the official views of the National Institutes of Health.

## Conflict of Interest

The authors declare that the research was conducted in the absence of any commercial or financial relationships that could be construed as a potential conflict of interest. The reviewer HS declared a past co-authorship/collaboration with one or more authors MF and AB.

## Publisher's Note

All claims expressed in this article are solely those of the authors and do not necessarily represent those of their affiliated organizations, or those of the publisher, the editors and the reviewers. Any product that may be evaluated in this article, or claim that may be made by its manufacturer, is not guaranteed or endorsed by the publisher.
